# Coarse-Grained Model for Prediction of Hole Mobility in Polyethylene

**DOI:** 10.1021/acs.jctc.3c00210

**Published:** 2023-10-16

**Authors:** Mikael Unge, Hannes Aspåker, Fritjof Nilsson, Max Pierre, Mikael S. Hedenqvist

**Affiliations:** † NKT HV Cables, Technology Consulting, SE-721 78 Västerås, Sweden; ‡ Department of Fibre and Polymer Technology, Polymeric Materials Division, School of Engineering Sciences in Chemistry, Biotechnology and Health, KTH Royal Institute of Technology, SE-100 44 Stockholm, Sweden; § FSCN Research Centre, Mid Sweden University, 85170 Sundsvall, Sweden

## Abstract

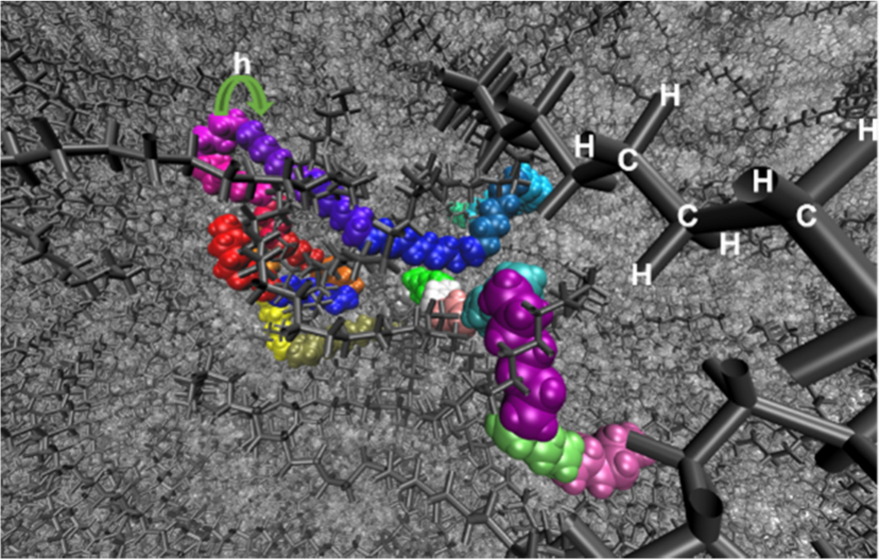

Electrical conductivity measurements of polyethylene indicate that the semicrystalline structure and morphology influence the conductivity. To include this effect in atomistic charge transport simulations, models that explicitly or implicitly take morphology into account are required. In the literature, charge transport simulations of amorphous polyethylene have been successfully performed using short oligomers to represent the polymer. However, a more realistic representation of the polymer structure is desired, requiring the development of fast and efficient charge transport algorithms that can handle large molecular systems through coarse-graining. Here, such a model for charge transport simulations in polyethylene is presented. Quantum chemistry calculations were used to define six segmentation rules on how to divide a polymer chain into shorter segments representing localized molecular orbitals. Applying the rules to amorphous systems yields distributions of segments with mode and median segment lengths relatively close to the persistence length of polyethylene. In an initial test, the segments of an amorphous polyethylene were used as hopping sites in kinetic Monte Carlo (KMC) simulations, which yielded simulated hole mobilities that were within the experimental range. The activation energy of the simulated system was lower compared to the experimental values reported in the literature. A conclusion may be that the experimental result can only be explained by a model containing chemical defects that generate deep traps.

## I Introduction

In the transition from fossil-based energy to a fossil-free energy society, various challenges follow, in particular, for some renewable energy sources such as off-shore wind and large-scale solar parks. Not seldom, the optimal place for these installations is far from where the energy is needed; naturally, off-shore wind farms are placed far from the shore and desert areas and may serve as places for large solar cell parks. To transmit the power to populated areas, high-voltage direct current (HVDC) cables are preferred in order to limit transmission losses. The HVDC cables operate at a high temperature due to joule heating in the conductor. Today, 70 °C is the maximum conductor temperature used in HVDC cables. If the conductivity of the insulation is too high, then the resistive heating leads to a “thermal runaway” and electrical breakdown. Thus, the design of the cable, in principle, the thickness of the insulation, relies on a balance between the electrical conductivity, thermal conductivity, and the resistance to electrical breakdown. Strategies to improve the cable endurance involve a decrease in electrical conductivity and an improvement in the resistance to electrical breakdown. State-of-the-art polymer cable insulation is based on polyolefins that have an electrical conductivity on the order of 0.1–10 fS/m at 30 MV/m and a temperature of 60–70 °C.^1−8^ To achieve these low conductivity levels, a first approach is to keep the materials clean from volatile components that could contribute as ionic charge carriers. With almost only electronic charge carriers, electrons, and holes, the strategies to further reduce conductivity are primarily to add nanoparticles or molecular additives. These additives are selected based on their charge trapping properties.^2,4,9,10^ A recently highlighted third approach is to take into account the semicrystalline nature of polyolefins and control the morphology.^1,3,11^ Thus, there is a need to further understand the basic underlying charge transport mechanisms in polyolefins, e.g., the impact of the morphology of the polymer.

Simulating electronic charge transport in organic materials is a challenging task. However, multiscale simulation approaches have been developed and applied successfully to a wide range of oligomers and polymers, in particular, conducting and semiconducting materials^12−19^ and, recently, dielectric materials.^20−24^ The electronic charge transport mechanism dominating in a particular material depends on the electronic structure properties and structural changes due to the excess electrons or the holes and, of course, if the material is disordered or crystalline. Assuming a disordered material, a key question is whether the electronic states close to the band edges are localized or delocalized. If delocalized, hole or excess electron states may still have strong electron–phonon coupling where a geometrical distortion follows the charge, as a so-called polaron. If the interaction is weak, then charge transfer will depend on the electronic structure bands (band conduction). For dielectric polymers, in particular polyethylene (PE), it has been shown that the states closest to the band edges are localized and that the mobility edge and activation energies are much larger than the thermal energy at relevant temperatures in both crystalline and amorphous regions.^25−29^ The electron mobility edge in PE is 0.3–0.4 eV away from the conduction band edge, and the hole-mobility edge is 0.2–0.3 eV away from the valence band edge, in good agreement with experimental values.^30−32^ This is in contrast to many semiconducting and conducting polymers where extended states may exist close to the band edge, and for such cases, the charge transport can, e.g., be bandlike or involve long-range hopping.^14−17^ For PE, effective mobility edges were calculated, including all accessible states, due to thermal energy. The effective electron- and hole-mobility edges were as low as 0.07 and 0.01 eV, respectively, which could be understood considering the high density-of-states close to the band edges in PE.^29^ However, in a real sample, vinyl groups and bonding defects, e.g., carbon double-, conjugated double-, and triple bonds, may occur along the chain. Additionally, some oxidation of the chains also is likely to occur. All of these “chemical” defects create electron and hole trapping sites with a depth of 0.3–1.8 eV, compared to PE band edges.^33^ Thus, it is expected that simulation of pure PE systems will yield lower activation energy compared with experimental results.

Also, in the case of localized charge carriers, the electron–phonon interaction can be weak or strong. If the interaction is weak, the charge transport can be described by the Miller–Abrahams hopping model, where the driving force between the sites, in energy terms, ΔG*, is entirely given by the difference in site energies.^34^ In an amorphous material, the transition rate could then also depend on the distance between the sites. In the last category of charge transport mechanisms, the interaction between the localized states and phonons is strong, and this interaction needs to be included in the evaluation of ΔG* of the charge transfer. In the Marcus theory of charge transfer, the changes in free energy are evaluated by the reorganization of the reactant and the product separately.^35^ This has been described in numerous publications; in summary, the Marcus theory describes hopping rates based on a few properties: site energy (*U*), reorganization energy (λ), and transfer integral (*J*) between two sites (see eq 1 below).^35^ If the reorganization energy is much larger than the transfer integral between the two sites, there is a strong electron–phonon interaction, and thus, the Marcus theory is an appropriate model for describing the charge transfer. For polyethylene, the relevant properties have been calculated and evaluated carefully in comparison with Fermi’s golden rule.^36,37^ The reorganization energies are much larger than the transfer integrals, and the typical vibration modes of the C–C bonds are on the order of 100 meV. However, this may violate the classical limit where the Marcus theory is valid, but compared with Fermi’s golden rule, the Marcus theory is concluded to be appropriate for a disordered PE system.^36^ The evaluation has been done for both intra- and interchain charge transfer. In summary, in polyethylene, the electronic states are localized, the mobility edges are far away from the valence band edge, and the electron–phonon interaction is strong. Hence, the Marcus theory of charge transfer is a reasonable model for polyethylene.

The properties needed in the Marcus theory are possible to determine from density functional theory (DFT) calculations, whereas the morphology of the system can be obtained from molecular dynamics simulation (MD) or Monte Carlo simulations.^38,39^ When the Marcus parameters are determined for a particular morphology, the mobility of the system can be determined using kinetic Monte Carlo (KMC) simulations.^19^ The KMC approach has primarily been used to simulate mobility in amorphous polymer structures where oligomer models are applicable. However, to simulate semicrystalline polymers and the effects of morphology, much longer polymer chains are required. In several studies, it has been shown that the specific morphology affects the conductivity and mobility of PE.^1,3,11,40,41^ Thus, it is important to be able to include the impact of morphology in the mobility simulations, which requires more realistic lengths of the polymer chains.^11^

**Figure 1:**
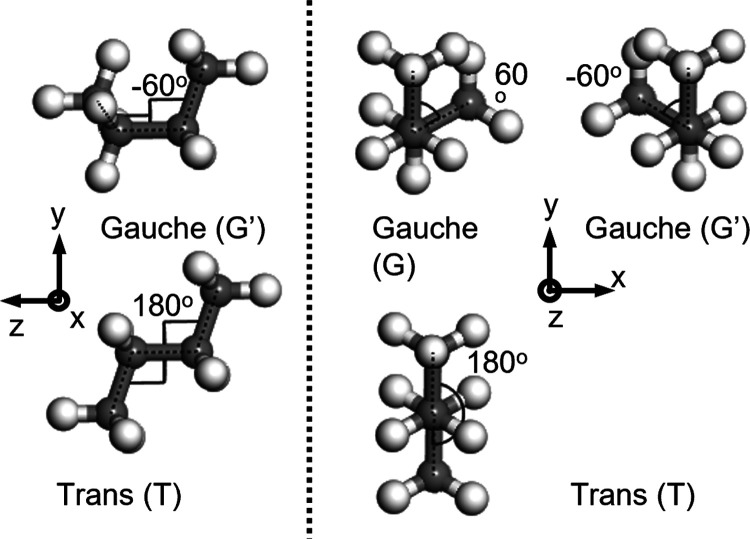
Illustration of trans and Gauche conformations of alkane, here, *n*-butane. Carbon atoms are in gray, and hydrogens are in white.

To compute the intrachain hopping of amorphous or semicrystalline polymer systems using KMC, the electronic states that localize along the polymer backbone must first be determined. In π-conjugated polymers, electron localization occurs as a consequence of rotation of torsion angles so that the π-orbitals become nonoverlapping. This can be used as a structural criterion since the π-orbitals are perpendicular to the molecular plane,^42^ although recent studies indicate that π-conjugated polymers are more complex than anticipated.^43^ However, dielectric polymers such as polyethylene and other polyolefins lack π-orbitals, and therefore, another strategy is necessary to identify how electronic states localize along the polymer backbone. Recently, an approach was proposed where the intrachain electronic state localization in polyethylene (PE) was correlated to the Kuhn length.^23^ However, this gives only an average length of the orbitals and is thus primarily suitable for oligomer models of the polymer, which can be used to study polymers in the amorphous phase. Localization criteria may also be determined from torsion-angle configurations along the polymer chain; see Figure 1. The torsion angles along the polymer backbone of PE have three energy minima, the global trans configuration (T, 180°), which is the global energy minimum, and two local minima, the Gauche and anti-Gauche configurations (G and G′, ±60°), which are local minima. By revealing how the electronic state localization of an ethylene oligomer is affected by the torsion angles along the backbone, an initial localization criterion of two adjacent G configurations is suggested.^44^ In amorphous PE, several additional configurations may lead to electronic state localization, and the purpose of this paper is to find a complete set of criteria that describe this. In this study, six rules for electronic state localization in PE are defined, and then, these are used to build a coarse-grained model for hole-mobility simulations in amorphous PE using the Marcus theory and KMC simulations.

## II Methods

### II.I Generation of the Amorphous Polymer Structure

Geometries of the amorphous structure of polyethylene were prepared and relaxed using molecular dynamics (MD) in GROMACS version 2022.^45^ The polymers, with 10–20 chains with 192 carbon atoms per chain, were initially packed in a large box with sides of 80 nm.^46^ An initial geometry optimization was performed followed by dynamic simulations using the OPLS-AA force field. A time step of 1 fs was used for solving the equation of motions using the Verlet algorithm, and a cutoff of 1.0 nm was applied to the nonbonded van der Waals interactions. The simulations were performed in the canonical ensemble (NVT) or isothermal–isobaric ensembles (NPT), using the Berendsen barostat and thermostat with a time constants of 2.0 ps.^47^ Electrostatic interactions were treated by the particle mesh Ewald (PME) method, with the reference pressure set to 1 bar. Initially, the simulations were performed for 2 ns at 600 K with constant pressure (NPT) followed by 5 ns with a constant volume (NVT). This was done in order to allow the polymer chain to stretch over the periodic boundaries since the initial packing was done without periodic boundary conditions. Then, the system was quenched down to 300 K and run for 5 ns with constant pressure (NPT) to reach the correct density. The densities of the final structures were around 0.85–0.86 g/cm^3^, in agreement with experimental values.^48^

### II.II Localization of Electronic States

To determine localization criteria for electronic state localization, different levels of quantum theory were applied on the calculation of orbitals of the model molecule representing a PE polymer chain. Both Hartree–Fock (HF, see, e.g.,^49^) and DFT with exact exchange (B3LYP)^50,51^ and in some cases without exact exchange (BLYP) were used.^52,53^ The BLYP exchange correlation functional was used for calculation of electronic states of a single full-length polymer chain from the amorphous PE structure. HF and DFT may predict localization differently, where the latter has a tendency to yield less localized states. All DFT calculations include dispersion corrections according to the TS scheme.^54^ Both HF and DFT calculations were performed in DMol3 using the DNP basis set, as implemented in BIOVIAs Materials Studio 2019.

Two oligomers, C_16_H_34_ and C_30_H_62_, were used as polyethylene models to study the impact of different combinations of torsion angles. The smaller oligomer was used for the simplest configurations, and the longer one was used for more complex configurations. After a configuration was specified, the oligomer was relaxed with molecular dynamics using the universal force field (UFF), and the resulting geometry was used for initial single-point HF and DFT calculations. Then, geometry optimization was performed using HF and DFT, separately. In many cases, the HF yielded data that did not converge; consequently, a final single-point HF calculation was performed on the last geometry from the geometry optimization. A single-point HF calculation was also performed on the DFT-B3LYP-TS optimized geometry. Localization of the orbitals was analyzed for all of these cases.

**Figure 2:**
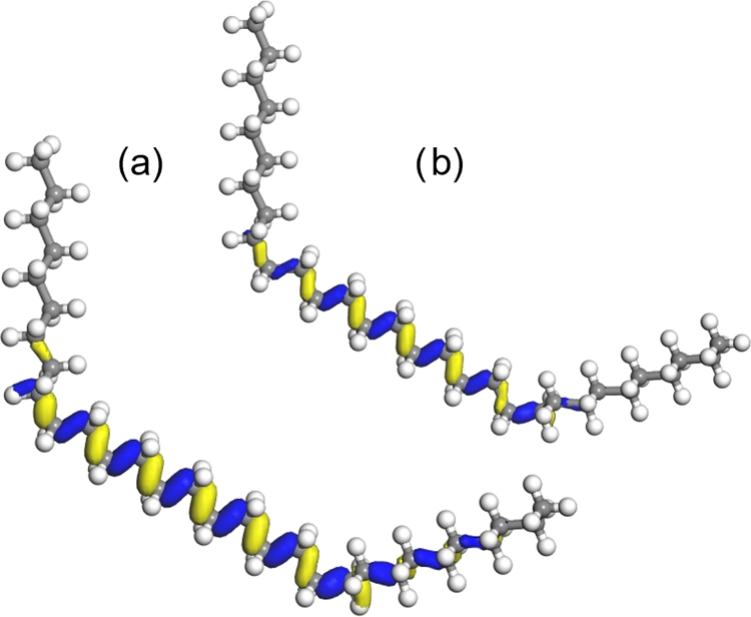
Example of localization of orbitals: the orbital is approximated to be localized to the part with the largest probability in the model. (a) HOMO with the iso value set to 0.06. (b) HOMO with the iso value set to 0.04.

Localization of orbitals was determined by visual inspection with the isosurface value set to 0.06. A smearing function for orbital occupation was used for improved convergence, and the activation energy was set to 0.005 Ha (∼0.14 eV). This led to a more diffuse effect of the localization criteria; therefore, the orbitals were defined to be localized to one side of a specific configuration of the polymer structure if the major part of the orbital was localized on that side. An example is shown in Figure 2a, where the HOMO was plotted with iso value 0.06, and there are components of the orbital on both sides of the two twists of the chain. However, in the analyses of the localization, the orbital was approximated to be localized to the section between the twists in the chain, which was motivated by the fact that the major part of the orbital was localized to this part of the chain. This is illustrated in Figure 2b, where the orbital is shown with a lower iso value and clearly only occupies the middle section of the chain. In a complex polymer configuration, an orbital can localize to both sides of a midsection (consisting of a few ethylene groups), and an adjacent orbital in energy also has the same feature (e.g., HOMO and HOMO-1). The orbitals were considered to be localized in this case, as well. This can be motivated by the high symmetry of the model polymer, which gives orbitals with similar energies for both segments of the model polymer. However, in an amorphous polymer, the symmetry would likely be broken both due to the actual configuration of the polymer backbone and due to effects from the surrounding polymer chains. Additional motivation to not be too strict in determining localization in this case is that the localization criteria should be used in defining the coarse-grained model for hopping transport along the polymer chain. The overlap and transfer integral along the chain will be large, thus resulting in a high hopping rate, in comparison with interchain hopping values where the transfer integral is much smaller. Hence, in mobility simulations, the number of jumps along the chains will be more important than the exact position of the jumps, i.e., how the chain is divided into segments.^42^ More detailed approaches like the inverse participation ratio (IPR) can be applied to investigate localization phenomena.^26,55^ Comparison with the IPR approach is discussed further in the results section.

### II.III Hopping Rates

Due to the localization of the electronic states along the polymer backbone, the charge transport can be described by a hopping process from one localized state to another localized state. Charge transfer rates are approximated by the Marcus rate expression^35,56^
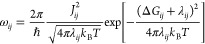
1where *T* is the temperature, *J*_*ij*_ is the transfer integral, λ_*ij*_ is the reorganization energy, and Δ*G*_*ij*_ is the change in the Gibbs free energy of the charge transfer between the sites. The parameters were calculated using DFT, and each determined segment of the polymer where electron localization occurred was treated as a separate oligomer and terminated with a hydrogen atom if needed. The transfer integral, *J*_*ij*_, between two localized states was calculated using the site-energy correction method.^57^ The localized states, from where the transfer integral should be calculated, are not generally orthogonal, which is assumed in the derivation of the charge transfer rate expression in eq 1. The site-energy correction method compensates for the nonorthogonality by taking into account the site energies and the overlap matrix, *S*_*ij*_ = ⟨ψ_*i*_|ψ_*j*_⟩, in the following way
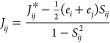
2where the uncorrected transfer integral is
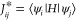
3and
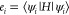
4

In determining the Gibbs free energy change, we include the internal energy difference of the sites that is evaluated from four molecular states using the expression
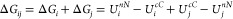
5where *U*_*i,j*_^*x,Y*^ is the total energy of the oligomer for site *i*, *j* for the neutral/charged (*x* = *n*, *c*) state in the neutral/charged geometry (*Y* = *N*, *C*). The reorganization energy, λ_*ij*_, is the change in the energy of a system due to its geometric relaxation upon charging or discharging. Also, the reorganization energy is calculated from molecular states as follows
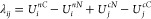
6

Since the segment lengths of the polymer can differ, the lengths of the model oligomers will also vary. Thus, the site and reorganization energies need to be calculated between all possible combinations of oligomer lengths that are relevant based on the obtained segments. A segment of the polymer backbone is restricted by larger geometrical changes due to bonding to the adjacent segments of the polymer backbone. By restricting geometrical changes by fixing the dihedral angles, unwanted relaxations of the model oligomer can be avoided. This was investigated by Sato et al.,^23^ and only minor changes were observed in simulated mobilities. In the present study, we do not include any restrictions in the oligomer optimization. In order to limit the number of combinations, only oligomers with all dihedral angles set to the trans configuration were considered.

All DFT calculations were done using NWChem^58^ with the B3LYP functional with the DZVP basis set. Effects from the surrounding polymer chains were taken into account by using the COSMO model with the dielectric constant set to 2.25.^59^

### II.IV Charge Mobility

Charge transport was simulated using the kinetic Monte Carlo (KMC), with periodic boundary conditions implemented in an in-house code; an introduction to KMC simulations is, e.g., given by Jansen.^60^ A single charge carrier was used and motivated by the low charge concentration in the investigated systems. Since a single charge carrier was used, the master equation to solve is
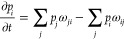
7where *p*_*j*_ is the probability for the charge to occupy site *j* and ω_*ij*_ is the Marcus hopping frequency. The hole mobility, μ, was calculated directly from the KMC trajectory as μ = *x*/*Et*, where *x* is the traveled distance in the field direction, *E* is the absolute value of the field, and *t* is the simulated time. The simulation was performed until the hole had traveled a distance that was 20 times the simulation cell in the electric field direction. From initial runs, this stopping criterion was seen to give good convergence for most cases; see more discussion below for some specific cases. Statistics were obtained from a large set of runs (45,000), where the initial site of the hole varies.

**Figure 3:**
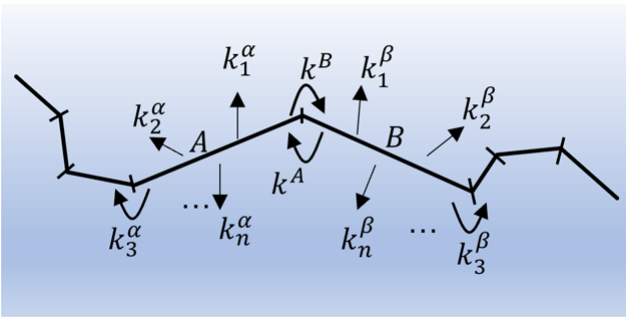
Illustration of two adjacent sites A and B along a polymer chain, forming a duplex. The hopping rates *k*^*A*^ and *k*^*B*^ are much higher compared to the other hopping rates *k*_*i*_^α^ and *k*_*i*_^β^, *i* = 1···*n*.

The different segments have a large variation in the site energy, but for specific combinations of sites, the charge can be temporarily trapped such that it jumps back and forth between two sites, which can significantly increase the simulation time. This can happen along the polymer backbone, in particular, for two long adjacent segments. In simulations with only one charge carrier, this phenomenon can be avoided by calculating the probabilities for which direction it eventually will jump.^61^ Consider two strongly interconnected sites A and B, in this case, two adjacent segments along the same polymer chain, as illustrated in Figure 3. The two sites are connected to each other via large hopping rates *k*^*A*^ and *k*^*B*^, and the two sites form a duplex where the charge is expected to spend a long time due to the high hopping rates between them. However, the duplex is also connected to additional sites α_*i*_ and β_*i*_, and these sites are both adjacent sites along the polymer chain and sites at nearby polymer chains. The hopping rates from one of the two sites in the duplex to nearby sites are much lower, *k*_*i*_^α^ and *k*_*i*_^β^ for sites A and B, respectively. If both *k*^*A*^ and *k*^*B*^ are large, the time that the charge hops back and forth between A and B will be long and would significantly increase the simulation time. As an example, if the hopping probability between two strongly connected sites is 99.9%, the number of jumps before the charge leaves the duplex would be on the order of 500 jumps. A way to improve the performance is to derive expressions for the probability of leaving the duplex rather than the two individual sites and thus avoid simulating all of the round trips done within the duplex. The escaping probability from the duplex from site A can be expressed as
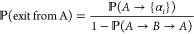
8and the expected number of round trips before leaving via site A as
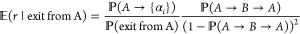
9Using (eq 8) in (eq 9) simplifies the expression to
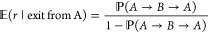
10The expected time the charge stays inside the duplex is calculated from
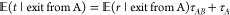
11where τ_*AB*_ is the time spent in the duplex and τ_*A*_ is the time spent at site A. The full derivation is included in the Supporting Information, including how τ_*AB*_ and τ_*A*_ are calculated and expressions for the escape via site B. Now, the probability of leaving the duplex can be determined from expression (eq 8) and the number of round trips and time from (eq 10) and (eq 11) instead of simulating all jumps within the duplex. The algorithm for the duplex escape probability and residence time in the duplex was implemented in the in-house code.

## III Results: Coarse-Grained Model

### III.I Review of the Double Gauche Localization Criterion

**Figure 4:**
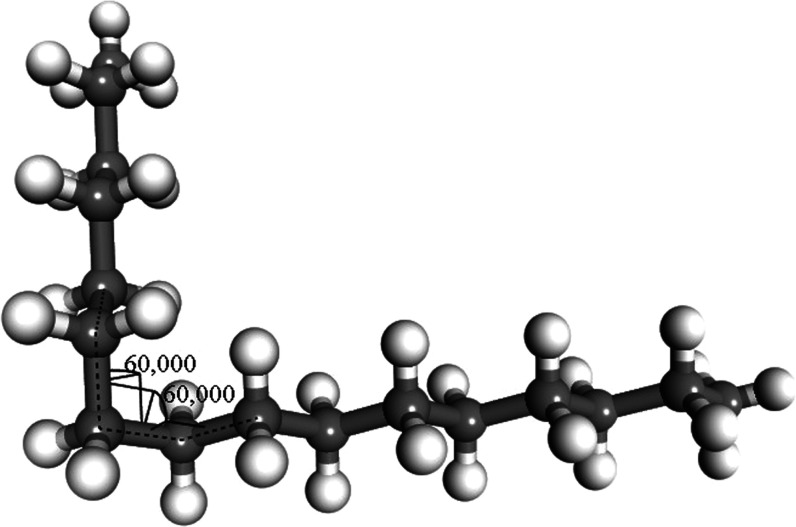
Example of configuration of C_16_H_34_ used in the calculations with two torsion angles that are not in the trans configuration, here in G and G′, respectively. Carbon atoms are in gray, and hydrogen atoms are in white.

Recently, a first result on localization criteria for valence states in PE was published.^44^ The criterion identified was identified based on DFT simulations of a PE model system, as described above. The model system used was an alkane chain with 16 carbon atoms, C_16_H_34_, in which different configurations were systematically examined with respect to valence state localization. An example of a molecule with two adjacent Gauche configurations is shown in Figure 4.

A summary of the simulation results is included in Table S1 of the Supporting Information. Three different geometries per configuration are listed, *Start*, *Initial*, and *Final*, where the first corresponds to ideal torsion angles with only T and G(G′) configurations with exactly 180° and 60°(−60°), respectively. The *Initial* geometry is after the initial geometry relaxation using MD with a simple force field, and the *Final* geometry is the HF/DFT optimized geometry. For the different geometries, the highest occupied molecular orbital (HOMO) and additional 2–3 orbitals were visually inspected.

From the result, it is clear that a single G conformation does not localize the orbitals. A small update compared to our previous work is included; previously, it was reported that a single G in the initial geometry did localize the orbitals.^44^ However, as will be explored below, if the oligomer is too short, the terminating methyl group will impact the states. To overcome the impact of the chain ends, a longer alkane chain with 30 C atoms was used to check the localization in the initial geometries, which with 16 C atoms did localize the orbitals. With the longer chain, the orbitals did not localize due to the single G configuration. By introducing adjacent Gauche configurations, G or G′, the orbitals localize to mainly one side of the double-G configuration; some orbitals stop at the first G/G′ and some stop at the second G/G′. The localization results were similar for both HF and DFT-B3LYP calculations. A single 192 C atom-long polyethylene chain was picked out from an amorphous PE configuration, optimized with molecular dynamics.^44^ This structure was then used in the DFT-BLYP simulation to see if the double-G configuration localizes orbitals in the polymer chain. Here, the double-G configuration occurs, and the states localize to either side of the configuration. However, it was also noted that two G/G′ separated with a T configuration (i.e., GTG, G′TG′, G′TG, or GTG′) also localize the orbital to either side. Hence, in a long polymer chain, many different configurations that could impact the orbital localization are possible. In the next section, we will investigate all possible configurations in amorphous PE and identify the needed criteria for how to cut a full polymer chain in segments representing the orbital localization.

### III.II Segmentation Rules

In order to define a set of criteria that define the localization of the orbitals in PE chains, quantum chemistry calculations were performed on a set of alkane chain configurations. Alkane chains with 30 C atoms were used. Two questions were stated to structure the configuration generation; (1) How does the orbital localize if there are more than two G states in a row? (2) How many T states are required between two G states before the G states are counted as two independent/uncorrelated G states?

For the first question, a set of structures was used for both HF and DFT calculations in the investigation of orbital localization, as described above. In order to limit the number of configurations for more than four G’s in a row, the assumption was made that both G and G′ yield the same impact, and only G was included in the considered configurations. Hence, we will not make a difference if, e.g., the configurations are GG, GG′, or G′G′, but treat all as GG configurations. This can be motivated from the first result presented above, where both G and G′ were included, but the localization behavior did not differ between the two. For three and four G in a row, combinations with both G and G′ are included in the set of configurations.

**Figure 5:**
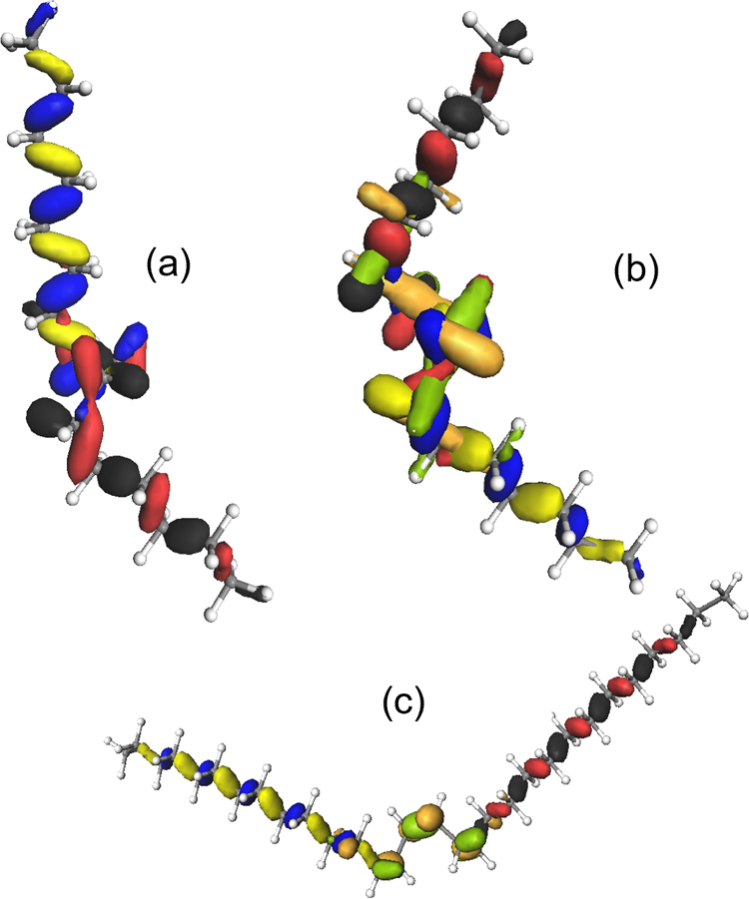
Orbitals from HF calculations. HOMO (blue-yellow), HOMO-1 (black-red), and HOMO-2 (orange-green), not included in (a), for different configurations of the midsection of the alkane chain. (a) TGGGGGT, (b) TGGGGGGT, and (c) TGGGGGGGT.

In Table S2, the localization behavior of the orbitals is summarized for configurations with multiple G states in a row. For 3–5 G states in a row, the results were similar as for double-G; the orbitals localize on either side of the G sequence with a cut approximately in the middle of the G sequence. With six or more G states in a row, up to 10 G states were included, and the states localized to the G sequence and on the sides. In Figure 5, three examples on how the orbitals look like are shown. For the case with five G states, the HOMO and HOMO-1 clearly localize on either side of the middle of the G sequence. All parts of the alkane chain are covered by the HOMO or HOMO-1 orbitals, i.e., any orbitals lower in the valence band do not cover any additional part of the chain and can thus be ignored. In the cases with six and seven G states in a row, respectively, it is clear that HOMO-2 localizes to a separate part of the chain, the G sequence. Hence, from these results and from the previous result of single and double-G states, the following rules can be defined: (1) a single G state does not impact localization of the state, (2) 2–5 G states in a row localize the state to either side of approximately the middle of the G sequence, and (3) six or more G states in a row localize the states to the G sequence and to both sides of the G sequence, respectively.

**Figure 6:**
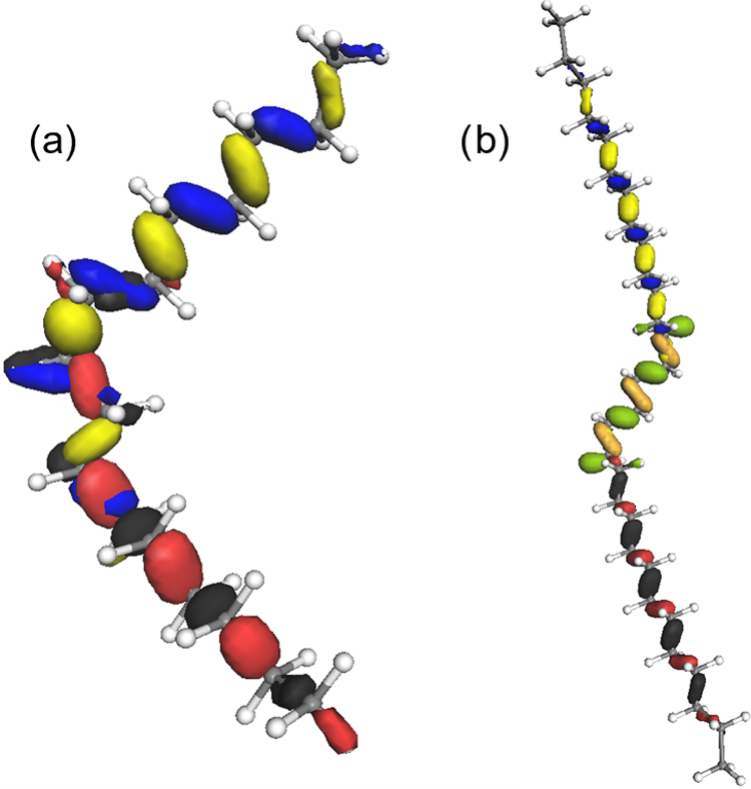
Orbitals from HF calculations. HOMO (blue-yellow), HOMO-1 (black-red), and HOMO-2 (orange-green), not included in (a) for different configurations of the midsection of the alkane chain. (a) TGTTGT and (b) TGTTTGT.

Now, we continue by investigating how the orbitals localize if one or several T configurations separate two G states to understand when the two states should be considered as a two-G configuration and a single G configuration, respectively. In Table S3, the results for this set of configurations are summarized. With just one or two T states separating the two G states, the orbitals localize to either side of the middle of the GTG and GTTG configuration, respectively. From three to six T states, the orbitals localize to the T configuration between the G states and before and after the entire configuration. From seven and more T states between the G states, the orbitals delocalize, i.e., from seven T states, the two G’s should be considered as single G states. In Figure 6, orbitals for the cases with two and three T states separating two G states are shown. It is clear that in the 2T case, the two orbitals cover the whole alkane chain and that they approximately localize on either side of the middle of the GTTG configuration. In the 3T case, the orbitals localize to three different parts of the alkane chain, to the 3T states in the middle, and to the sides of the entire GTTTG configuration.

Figure S1 shows the cases with six, seven, and eight T separating the two G states. With six T states, it is observed that the orbitals localize to three separate sections of the alkane chain. However, for this configuration, there was already a tendency that HOMO and HOMO-1 localize to other parts of the chain, in addition to where they primarily localized, but HOMO-2 localizes to the midsection. With seven T, the trend that the orbitals localize to other parts of the chain becomes clearer, and HOMO-1 localizes to both sides of the 7 T configuration. In the eight T configuration, the HOMO was partly localized to all three parts of the chain. Here, the chain end can also affect the orbital localization in that part of the molecule. In summary, exactly how many T states that should be added between the two G states so that the HOMO delocalizes is a “moving” target, around six to eight T. In the present coarse-grained model, we set the rule that seven or more T states between two G states delocalize the orbitals. From this, we define two additional rules: (4) single or double T states between two G states localize the orbitals to either side of the middle of the configuration, with a single cut in the middle of the T sequence; and (5) three to six T states between two G states localize the orbitals to the midsection and to both sides of the G states, with two cuts before the two G states.

The cutting rules are implemented in a script that reads a PE structure and cuts the polymer chains accordingly. In the implementation, the segmentation rules are executed without any particular priority, which can generate cuts close to each other. Therefore, a sixth rule is applied: (6) if a segment becomes shorter than 4 T or G states, the two nearby cuts are removed, and a new cut is made in the middle of the old short segment. This extra rule is motivated by the fact that no orbitals have been observed that clearly localize to less than four CH_2_ units in any of the DFT and HF calculations. An additional motivation is that the rule is used in a coarse-grained model intended for hole-mobility simulations, and thus, the exact position of the cut is less important than the number of cuts. Note that rule 5 includes one G in the middle section; thus, the shortest segment becomes GTTT, and hence, rule 6 does not affect segments defined by rule 5.

**Figure 7:**
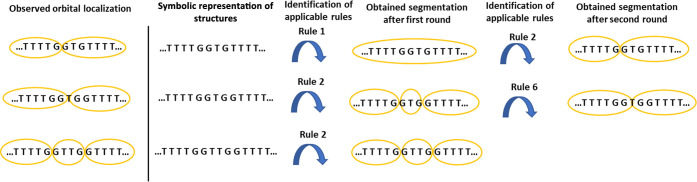
Three different configurations where the cutting rules have been applied. Left: Orbital localization as observed in HF and DFT calculations. Right: Symbolic representation of the structures and segmentation by stepwise applying the segmentation rules.

An initial test of the rules was performed on three investigated cases, TGGTGT, TGGTGGTT, and TGGTTGGT; see the illustration in Figure 7. From HF and DFT results, the orbitals localize so that a cut should be made in the double-G conformation, but no additional cuts in the first case (TGGTGT). In the second case (TGGTGGTT), there should be a cut in the middle of the sequence when compared to the calculated orbitals. After applying the initial impacting rule, Rule 2, there are three segments. The segment in the middle is too short according to Rule 6, and a new cut is made in the middle of the short segment and the previous cut is removed; the obtained segments from the rules then match the observed orbital localization. The third case (TGGTTGGT) represents two double-G configurations separated by two T states; using Rule 2 directly gives the correct segmentation in comparison with the observed orbital localization behavior. No additional rule impacts the segmentation.

**Figure 8:**
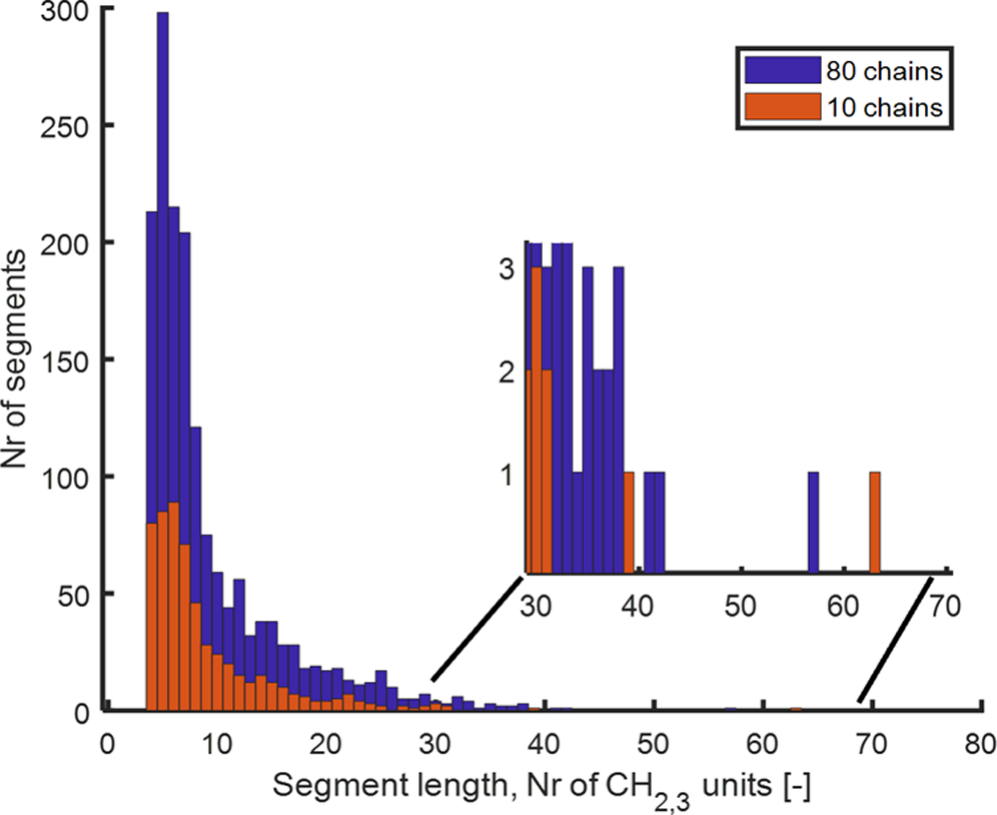
Histogram with the number of segments with a specific segment length in amorphous PE of the chain length of 192 CH_2,3_ units. The inset is a zoomed-in part, with the longest segments in the histogram.

This method is now applied on an amorphous PE structure containing 10 chains, each with 192 C atoms. In Figure 8, a histogram of the segment length distribution is shown. A majority of the segments had a length between four and six CH_2_ units, with five units as the mode (i.e., the most frequent number). Segments of up to 63CH_2_ were observed. On average, the segment length was 8.3 CH_2_ units, ∼10.4 Å. Another system with 80 chains of the same length was prepared in the same way and analyzed. In Figure 8, a histogram for the larger system is also shown, and the same trend was observed with a maximum length of 57 units. The mean value was 9.4 (11.8 Å) with a mode of 5 (6.3 Å) and with a median of 7 units (8.8 Å). The persistence length is a measure of the bending stiffness of a polymer chain. It is calculated from the autocorrelation of the bond angles along the polymer chain using the decaying exponential function.^62^ The persistence length is defined as half the Kuhn length and has been determined to be 6.5–9.0 Å in PE, depending on the amount of short-chain branching, where the shortest length is for linear PE.^62^ Thus, the mode and median segment lengths from the segmentation, following the proposed rules, were relatively close to the persistence length of PE. Hence, there is an indicative coupling between the structural properties and the electronic localization behavior.

In another work, localization of orbitals in an alkane chain was calculated using the IPR (inverse participation per monomer) method based on DFT-B3LYP results.^23^ In this case, it was concluded that the orbitals, on average, localize to 12 CH_2_ units ∼14 Å, which is close to the Kuhn length of linear PE, 13 Å.^42^ Thus, in comparison, the current approach yields shorter localization lengths. By using the IPR method, all C atoms that have some nonzero fraction of the electronic state are included in the IPR value. Thus, numerical noise and impact from exchange correlation functionals used may impact the result, where, e.g., DFT-B3LYP is expected to give more delocalized orbitals compared to HF. Relatively large fluctuations in the IPR value (up to 3 units) can be seen where the orbital continues or not after a twist in the backbone.^23^ It is likely that another orbital close in energy localizes primarily to the region with low probability of the first orbital; in this study, we excluded that region. Such an approach could be compared with an IPR approach using a threshold of which coefficients to include. Furthermore, we have also used HF in this study, giving more localized orbitals in comparison with DFT-B3LYP that was used in the other study. Additionally, the amorphous structure used in this study had a density of 0.856 g/cm^3^, to be compared with 0.83 g/cm^3^ in the other case. The difference was probably due to the different force fields applied. The higher density could introduce more twists of the polymer chain in order to pack it more efficiently, without crystallization, which then would lead to more cuts and segments according to the cutting rules defined in this work.

In Figure S2, a single chain from a 10-chain system is shown in two versions. The left version shows the segmentation performed by the method described above, dividing the chain into 22 segments. The other version shows the polymer and 19 orbitals obtained from a BLYP-TS calculation, where at least four additional orbitals would be needed to cover the entire polymer chain. Hence, the number of segments is in good agreement with the expected 22 orbitals to fully cover the polymer chain. When comparing where the orbitals localize versus the segments’ positions and lengths, the match is good but not perfect. In particular, at one end of the chain, marked with an arrow, the match is poor. However, by slightly changing the iso value of the blue-yellow orbital, it became localized to the middle of its current spread. The segmentation rules were defined by both HF and B3LYP-TS calculation results, and the result for the polymer chain is from BLYP-TS calculations, which can give too delocalized orbitals.

**Figure 9:**
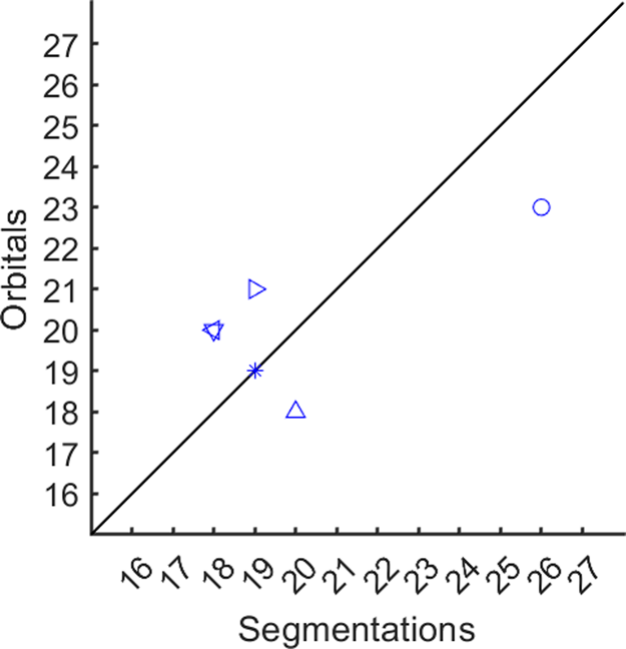
Number of segments per chain, as obtained by the segmentation rules versus observed number of orbitals from the DFT-BLYP-TS simulations. The chains were isolated from a fully amorphous system with 80 chains containing 192 carbon atoms each, which had been relaxed with MD simulations.

Figure 9 shows the number of orbitals needed to cover a full polymer chain versus the number of segments the rules provide. For this, six chains were taken out from the system with 80 chains and DFT-BLYP-TS calculation was performed on each chain. The required number of orbitals needed to cover the full polymer chains was determined by visual inspection. Of the investigated cases, a rather good prediction was seen: one case with a perfect match, four cases with a deviation of up to two segments (too many or too few), and one with three segments too few. The cases with too few segments could be due to the fact that cuts were made too close to each other, and that rule 6 was applied, i.e., that no segment should be smaller than four T or G states. Hence, rule 6 will reduce the number of cuts, if applied. For several cases, many Gauche states were in close proximity and therefore resulted in a chain with many twists; the orbitals in these regions overlapped quite much. The difference in energy levels is higher, 0.10–0.42 eV, compared to the average difference between orbital *n* and *n* + 1, 0.03 eV. Hence, it is likely that only one of the two partly overlapping orbitals will be needed to describe charge transport along that part of the chain. Additionally, for one case, a long segment, 38 monomers, was also noticed to be represented by three orbitals, i.e., two additional cuts should have been done. Analyzing this part of the chain, it was observed that single G states separated by more than seven T states still could be a point where the orbital localizes. Hence, probably a more complex rule is required to define when a single G state should not localize, e.g., including how the segments end in terms of G and T states. Furthermore, the model only includes one orbital per segment, which could impact the kinetics. However, since the charge carrier density is low, only one charge carrier was included in the simulation, the kinetics will not be impact due to only one orbital per segment.

The physical reason as to why the localization occurred the way it did in the identified T and G configurations is not trivial. Along the chain, the potential origin from both atomic potentials and the dispersive forces yields the potential that determines the T state of the dihedral angle to the global energy minima of an alkane chain, as well as the local minima, i.e. the two Gauche states G and G′. A hypothesis is that when the dihedral angle deviates more from the ideal T and G states, the potential energy increases in these sections, leading to the orbitals relaxing to either side of such a configuration. By calculating the dihedral angle deviations along three chains, an increased deviation from the ideal dihedral angles is observed around where the orbitals stop and the segment cuts, indicating that the potential energy is higher in these regions. Some larger deviations are also observed in the middle of long segments, so a large deviation is not a sufficient requirement but perhaps a necessary requirement.

An important question is whether the present set of rules is sufficient to describe the complete system, i.e., are there configurations not covered by the present set of rules. Primarily, that would be, as discussed above, a more detailed requirement on when G states should be considered single G states and sequences of seven G states or longer. In the amorphous system studied above, such long configurations of the G states were not detected. In combination with the analysis of correlation between the number of segments and the number of orbitals needed to cover one polymer chain, it is likely that the set of rules is sufficient but could be refined.

**Figure 10:**
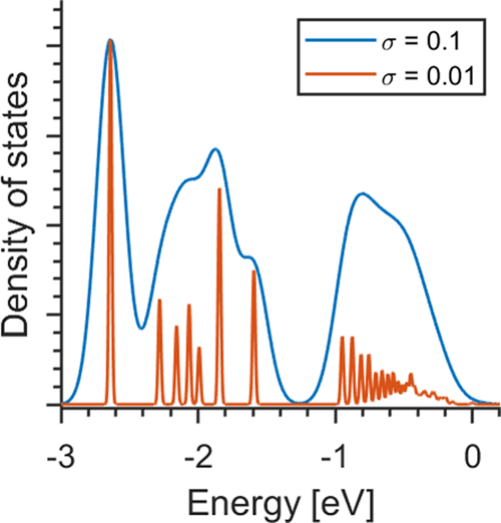
Density of states of the valence band of an amorphous PE with 80 chains with length 192 CH_2,3_ units. σ is the standard deviation of the gaussian functions.

A density of states (DOS) was calculated based on the segments obtained from an amorphous PE system with 80 chains. Using isolated oligomers with corresponding lengths of the segments from the amorphous PE, site energies were calculated with B3LYP DNP. A Gaussian function was added around each site energy to obtain a DOS (Figure 10), where the zero level was aligned with the site energy of the longest segment. Closest to the band edge, the states are close to each other, and even with narrow Gaussian functions, the bands are connected/continuous. Deeper in, the band states are more separated and correspond to shorter segments. The DOS indicates that the transport primarily will take place between the longer segments and that the short segment may act as blocking centers along the chains.

The proposed method to find the localization criterion can be applied to other polymer systems with clearly localized orbitals, i.e., where the energy gap between the mobility edge and the band edges is much larger than the thermal energy. This is expected to be the case for polypropylene (PP) and branched versions of PE and PP, and new rules are likely required.

## IV Results: Mobility Simulations

As a first test of the coarse-grained model based on the six segmentation rules, we simulate hole mobility in amorphous PE using the Marcus theory. Below, we describe how the necessary parameters are obtained and the mobility results are presented.

### IV.I Site Energies, Reorganization Energies, and Transfer Integrals

Before the KMC simulations, the different properties needed in the Marcus expression for the hopping rate needed to be determined. Since there will be different lengths of segments, the results for reorganization energies and transfer integrals will be in the form of a matrix for charge transfer between a segment with length *n* to a segment of length *m*, where *n* and *m* can take values from 4 and higher. In order to reduce the number of variations of segments, all segments of a specific length were represented by an oligomer with the same length, but all dihedral angles were in the trans configuration.

**Figure 11:**
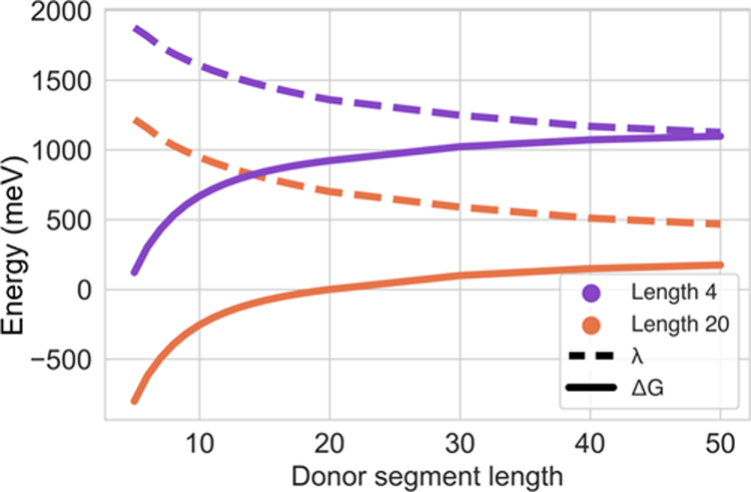
Reorganization energy, λ, and site-energy difference, Δ*G*_*ij*_, for hole transfer from donor segments of increasing length to acceptor segments of length 4 or 20.

First, the site energies and reorganization energies were calculated according to the four-point method as described above; see eqs 5 and 6. The four different state energies were determined from DFT calculations of all-trans configurations of the oligomers. Figure 11 shows site-energy differences, Δ*G*_*ij*_, and reorganization energies, λ, as a function of the length of the donor segment for two acceptor lengths. The calculated reorganization energies decreased with a longer acceptor length, which is in agreement with the reported results.^7^ The calculated reorganization energies with donor lengths of 10–20 carbon atoms were in the range of ∼700–1180 meV. The site-energy difference, Δ*G*_*ij*_, is negative when the donor length is shorter than the acceptor length, i.e., it is energetically favorable for the hole to jump from a shorter to a longer segment. The standard deviation of the site-energy differences for donor lengths 10–20 was 126 meV, which is in good agreement with the literature data of 150 meV.^23^ This relatively small difference indicated that the approximation of only using all-trans oligomers is acceptable. In the case of charge transfer from two segments of equal length, the site-energy difference would be zero in the model. To avoid this, a Gaussian distribution can be added to each pair of sites to mimic naturally occurring variations in a system. These variations can be due to the surrounding polymer chains and Gauche states along the original segment. In this study, a Gaussian offset was added with a standard deviation of 7 meV unless otherwise stated. With the offset of 7 meV, the standard deviation of site energies of segments of length 10–20 was 150 meV, which is in agreement with a calculated distribution that included all unique configurations of the PE oligomer system.^23^ For all of the systems, the standard deviation was 455 meV, which is due to the fact that a significant number of segments is shorter than 10 or larger than 20. In a similar way, an offset from a normal distribution with standard deviation 100 meV was added to the reorganization energies to take into account observed variations in amorphous dodecane.^23^

The site-energy correction method was used for calculating the transfer integrals, *J*_*ij*_, between the segments in a polymer system. Each segment will have one or two unbound valence electrons from the cutting at the terminating carbon atoms; hence, hydrogen atoms were added in a 3-fold rotational symmetry in relation to the two already existing hydrogen atoms. The segments were neutral in the calculations, which, together with the site-energy correction method, have been shown to give reasonable results.^23^ For the transfer integrals between interchain sites, a cutoff was introduced to limit the number pairs to evaluate. The cutoff, set to 5 Å, was evaluated for the minimum distance between any two carbon atoms in the two segments. The cutoff distance was chosen based on a study where the convergence in simulated hole mobility was observed for a cutoff of 4 Å.^23^ Mean transfer integrals between all interchain sites were 4.1 meV, which is close to a reported value of 5 meV.^37^ For intrachain transfer integrals, a constant value of 50 meV was used for jumps within the chain.^37^ Below, the results of a small parameter study to evaluate the value of the intrachain transfer integral on the final results are presented.

### IV.II Kinetic Monte Carlo

**Figure 12:**
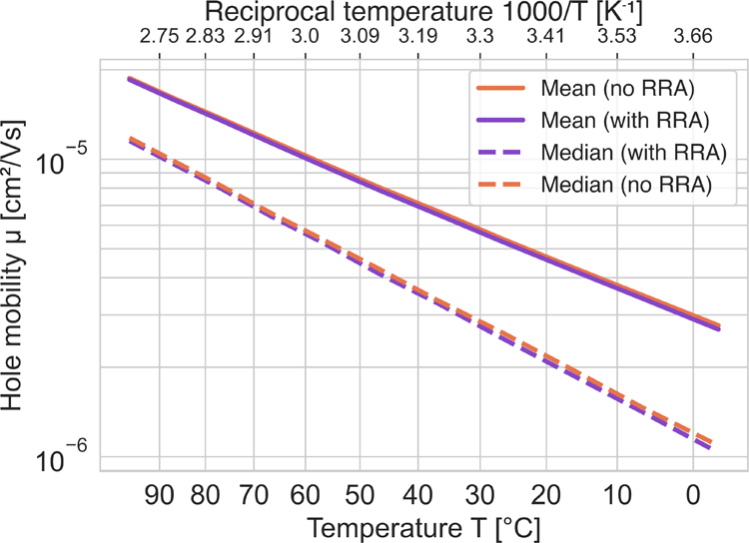
Mean and median hole mobilities with and without the use of the RRA algorithm.

The developed model for determining the hopping sites was applied to a set of five different amorphous PE systems, where the voltage was applied from all sides of the box and five different random offsets were used. Hence, a total of 150 different cases were used in the study. Each polymer system had 20 chains with 192 CH_2_ units each, and the calculations were performed at different temperatures. The density variations of PE within the applied temperature range were small, and the same structure was therefore used for all temperatures. First, the impact of the duplex escape algorithm, the round-trip reduction algorithm (RRA), was evaluated. In Figure 12, the mean and median of simulated hole mobilities are shown as a function of temperature, both with and without using the RRA. Both the median and mean values were overlapping. In the present result, 0.8% of the runs without RRA terminated before the hole had traveled a single box length and 26% of the trajectories stopped due to the set maximum number of steps. With RRA, only 0.045% of the trajectories stopped before a convergence in the mobility was reached and only 0.00037% stopped since the maximum number of steps was reached. For the largest studied intrachain transfer integral of 500 meV, see the parameter study below, it was not possible to reach convergence without RRA with the chosen step limit; the reason was that 29% of all hole trajectories terminated before traveling even a single box length. In comparison, the same statistics with RRA enabled was only 0.22%. This can be understood from the fact that without the RRA the hole remained at duplexes during most of the jumps in the KMC. Since a maximum number of jumps of 200,000 was used, the KMC run may stop before reaching a stable mobility value. This can give high mobility values if the expected time the charge carrier should spend in the duplex is not taken into account in the simulation. The slightly higher mobility values observed without using the RRA could be explained by this. In conclusion, the RRA significantly reduced the simulation time to reach convergence in these systems.

Experimental mobility values for PE are reported in a wide range, from 10^–10^ to 10^–7^ cm^2^/(Vs) depending on the measurement method and PE type.^31,63,64^ Low-density PE (LDPE) has a higher mobility than high-density PE (HDPE), up to a factor 100 for 1 mm thick samples.^63^ This indicates that 100% amorphous PE (as in the simulations) would have mobility values at the higher end of the range or even higher. For thin films, the mobility values are on the order 10^–7^ cm^2^/(Vs) for both HDPE and LDPE.^31^ Thus, the simulation results are higher than expected. It has previously been shown that introduction of trapping sites via carbonyl groups can reduce the mobility by orders of magnitude, which can explain the difference between the simulated and the experimental values where different types of trapping impurities may impact the experimental results.^20^ The trap states, due to chain defects containing carbonyl groups, carbon double bonds, or chemicals/impurities with similar chemistry, have a depth of around 1 eV.^33,65,66^ Additionally, it has been shown that finite size effects due to the limited system size in the simulation overestimate the mobility.^67^ If the simulation is performed on a semicrystalline PE, which would be similar to LDPE, instead of 100% amorphous PE, it is expected that the mobility would decrease. Since the crystalline phase has a larger band gap, the charge carrier is expected to follow the amorphous phase in the semicrystalline structure.^11,29^ It is reasonable to compare the increased path for the holes in the semicrystalline morphology with simulation results of penetrant diffusion of small molecules following the amorphous phase in the semicrystalline morphology; for 50% crystallinity, the scaling factor is 0.2–0.3.^68^ In summary, compared to experiments, a factor of 0.01 may be applied to the simulated mobility to account for both morphology and impurities. Furthermore, the obtained activation energy was 106 meV, which is about half of the expected value of ∼200–250 meV.^26,29^ Introduction of deep trapping states would not only reduce the mobility as discussed above but also increase the activation energy. Hence, the result in this study shows that the experimental result cannot be explained by a purely intrinsic charge transport mechanism of pristine PE but needs to include defects/impurities to match both mobility and activation energies. Below, two initial parameter scaling studies are included.

**Figure 13:**
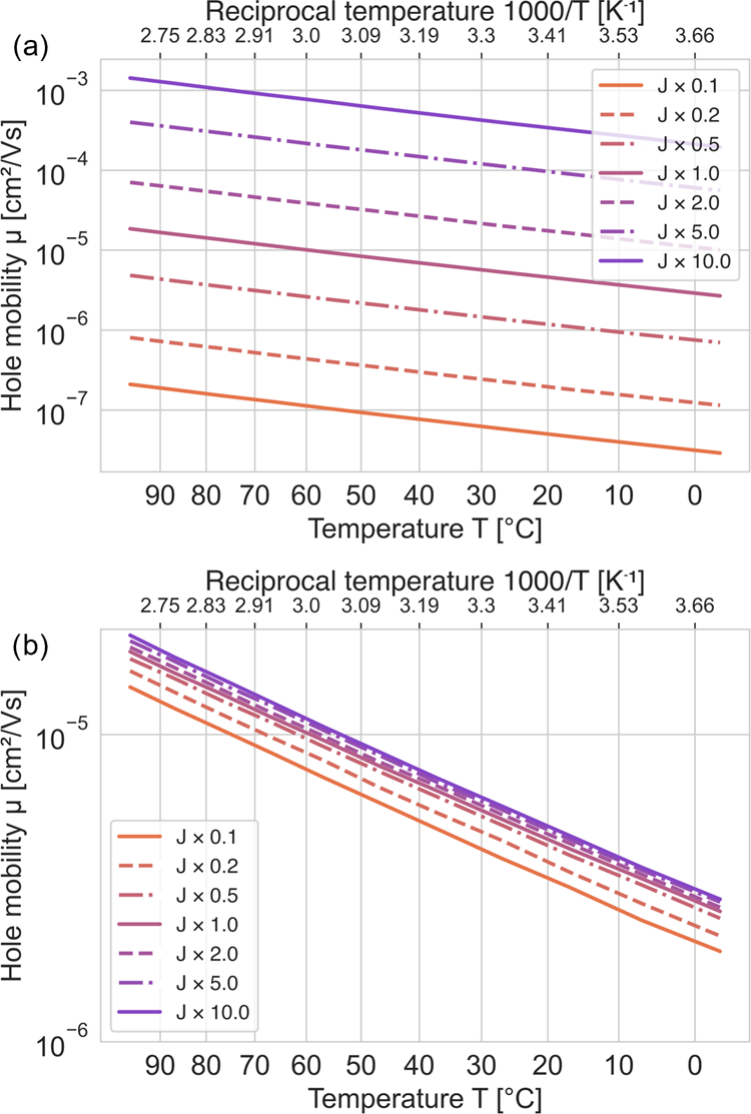
Simulated hole mobilities, μ, with different scaling factors applied to the transfer integrals (*J*). (a) Interchain and (b) intrachain transfer integrals scaled.

The parameters used in the hopping rate expression may also affect the resulting mobility values. Here, variations in two properties are reported, the transfer integral, *J*_*ij*_, and the energetic disorder (standard deviation), σ_*i*_, of the Gaussian function that was added to site energies *U*_*i*_. More parameter studies have been reported by Aspåker.^61^ From the results presented in Figure 13, it is clear that the interchain hopping is the limiting factor for the mobility values. Changes in the intrachain transfer integrals yield less impact on mobility. This may be counterintuitive, but as discussed by Sato et al.,^23^ when the distance traveled by the hole is longer than the oligomer/segment length, the limiting factor for mobility will be interchain hopping, which is clearly seen in our results. This is in line with the DOS for the system in Figure 10 where the deeper states from the short segment block the charge transport along the chain. Hence, it also means that the exact value of the intrachain transfer integrals is not that important when describing the charge mobility, at least for amorphous systems.

**Figure 14:**
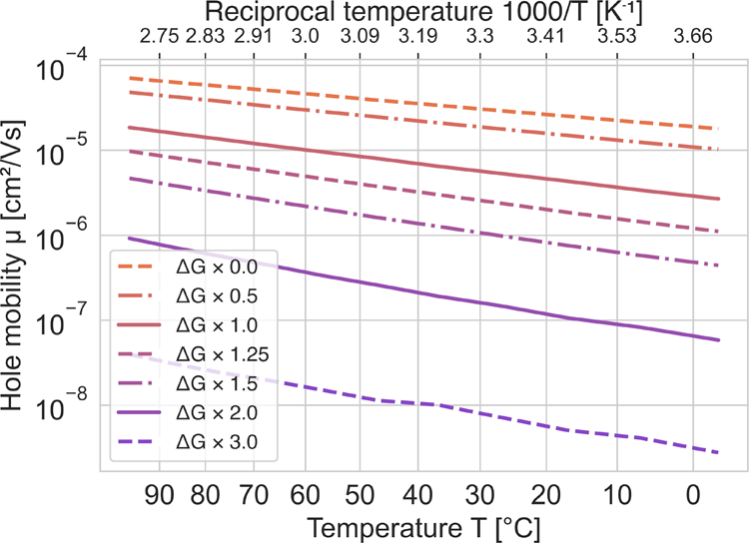
Simulated hole mobilities, μ, with different energetic disorders added to the site energies, Δ*G*.

A second parameter that is of interest to study is the standard deviation of the offset added to the site energies σ_*i*_. The scaling of the standard deviation was made so that the mean value remained the same for each segment length. The initial value used was 7 meV, which reproduced the site distribution for segments between 10 and 20 units long with a standard deviation of 150 meV. With all segments included, the standard deviation for the sites was 455 meV for all systems. Due to the approximation of using all-trans oligomers in the calculation, i.e., excluding Gauche states, and due to the effects from the surrounding polymer chains, there is an uncertainty on how large the offset should be. In Figure 14, results are shown from hole-mobility simulations, where the site-energy offset was varied. If the offset is excluded (σ = 0), the mobility increases by a factor of 5–8, depending on the actual temperature, but if the offset is increased instead, a reduction of the mobility was observed as expected due to the increased spread of site energies. The activation energy for the case of the largest offset disorder, Δ*G* × 3.0, was 243 meV, which is close to the experimental activation energy. Thus, the uncertainty in the offset value is a topic for further studies to increase the predictability of the model. As discussed above, a larger offset may originate from the presence of chemical defects/impurities.

## V Conclusions

We developed a coarse-grained model for hole mobility in polyethylene (PE). The model is based on KMC simulations, where the hopping is described by the Marcus theory. The sites along the polymer chains are defined using six torsion-angle rules, which were derived using quantum chemistry calculations of electronic states and correlated to the torsion angles along the backbone. Amorphous polyethylene was used to test the model, giving simulated hole mobilities in the upper range of the reported experimental values. This is reasonable since the experimental values were for semicrystalline polyethylene, which is expected to be lower due to the longer traveling path for the charge carriers circumventing the crystals. Compared to corresponding simulations of oligomer-based models, the hole mobility was a factor of 10 higher. This can be due to longer chain segments in the present model or due to uncertainties in some of the parameters. Also, the activation energy of the simulated system was lower compared to the experimental values reported in the literature; primarily, the added offset to site energies impacts this. A conclusion may be that the experimental result can only be explained by a model containing chemical defects generating deep traps. This will be further investigated in a future work.

The round-trip reduction algorithm (RRA) developed by Aspåker was shown to effectively reduce the simulation time for the system.^61^ With the RRA, it will be possible to use the model on larger systems where effects of the semicrystalline morphology of PE (and other semicrystalline polymers) can be addressed.

## Supporting Information Available

Tables S1–S3 and Figures S1–S3 are included in the Supporting Information, which also includes full derivation of the duplex algorithm (PDF)
