# Supplementary material for: Coarse-Grained Model for Prediction of Hole Mobility in Polyethylene
Source: J Chem Theory Comput. 2023 Oct 16;19(21):7882–94. doi: 10.1021/acs.jctc.3c00210 (PMC10653082; doi:10.1021/acs.jctc.3c00210)
Supplement: Supplementary file 1 — ct3c00210_si_001.pdf [file ct3c00210_si_001.pdf]

Supporting Information: Coarse-Grained Model for Prediction of Hole Mobility in Polyethylene

Mikael Unge[a,b*], Hannes Aspåker[a], Fritjof Nilsson[b,d], Max Pierre[b] and Mikael S. Hedenqvist[b]

[a] NKT High Voltage Cables, Technology Consulting, SE–721 78 Västerås, Sweden

[b] Department Fibre and Polymer Technology, Polymeric Materials Division, School of Engineering Sciences in Chemistry, Biotechnology and Health, KTH Royal Institute of Technology, SE-100 44 Stockholm, Sweden.

[c] Department of Mathematics, KTH Royal Institute of Technology, SE-100 44 Stockholm, Sweden.

[d]FSCN research centre, Mid Sweden University, 85170 Sundsvall, Sweden

* Corresponding author: Mikael Unge

E–mail address: mikael.unge@nkt.com , mikun@kth.se

I. SUMMARY OF ORBITAL LOCALIZATION CALCULATIONS

Here the full set of DFT and HF calculations done to evaluate orbital localization in alkane oligomers with 16 or 30 carbon atoms. The results are tabulated in in three tables S1, S2 and S3. The notation used for indication how the states localize use "|" sign to show where the orbital stop, on the side which doesn't have a "|" the orbital continues the full length of the remaining CH_2 units. Table S1 summarize the result for the cases with 2 adjacent G-states. Impact of the number of adjacent G-states on the localization is summarized in Table S2. Finally, Table S3 include the result from on how many T states are required between two G states before the G states are counted as two independent/uncorrelated G states

*Table S1 Summary of electronic structure calculations of $C_{16}H_{34}$. The notation used to describe where localization occur is described in the text. The numbers correspond to the two torsion angles. The two initial geometries marked with * are from a longer alkane chain with 30 C atoms.*

<table>
<tr><th rowspan="2">Method</th><th rowspan="2">Conformation</th><th colspan="2">Torsion angles</th><th></th><th></th></tr>
<tr><th>1</th><th>2</th><th>Localize?</th><th>Where?</th></tr>
<tr><td rowspan="3">HF</td><td>Start</td><td>60</td><td>180</td><td></td><td></td></tr>
<tr><td>Initial*</td><td>61</td><td></td><td>No</td><td></td></tr>
<tr><td>Final</td><td>63</td><td></td><td>No</td><td></td></tr>
<tr><td rowspan="2">B3LYP</td><td>Initial*</td><td>61</td><td></td><td>No</td><td></td></tr>
<tr><td>Final</td><td>95</td><td></td><td>No</td><td></td></tr>
<tr><td rowspan="3">HF</td><td>Start</td><td>-60</td><td>180</td><td></td><td></td></tr>
<tr><td>Initial</td><td>-61</td><td></td><td>No</td><td></td></tr>
<tr><td>Final</td><td>-62</td><td></td><td>No</td><td></td></tr>
<tr><td rowspan="2">B3LYP</td><td>Initial</td><td>-61</td><td></td><td>No</td><td></td></tr>
<tr><td>Final</td><td>-80</td><td></td><td>No</td><td></td></tr>
<tr><td rowspan="3">HF</td><td>Start</td><td>60</td><td>60</td><td></td><td></td></tr>
<tr><td>Initial</td><td>61</td><td>61</td><td>Yes</td><td>1|- |2</td></tr>
<tr><td>Final</td><td>63</td><td>49</td><td>Yes</td><td>1 | - |1 2</td></tr>
<tr><td rowspan="2">B3LYP</td><td>Initial</td><td>61</td><td>61</td><td>Yes</td><td>1| - |1 2</td></tr>
<tr><td>Final</td><td>55</td><td>55</td><td>Yes</td><td>1| - |1 2</td></tr>
<tr><td rowspan="3">HF</td><td>Start</td><td>60</td><td>-60</td><td></td><td></td></tr>
<tr><td>Initial</td><td>64</td><td>-64</td><td>Yes</td><td>1 |-| 2</td></tr>
<tr><td>Final</td><td>59</td><td>-79</td><td>Yes</td><td>1 2| - |1 2</td></tr>
<tr><td rowspan="2">B3LYP</td><td>Initial</td><td>64</td><td>-64</td><td>Yes</td><td>1 2| - | 2</td></tr>
<tr><td>Final</td><td>57</td><td>-88</td><td>Yes</td><td>1 2| - | 1 2</td></tr>
<tr><td rowspan="3">HF</td><td>Start</td><td>-60</td><td>-60</td><td></td><td></td></tr>
<tr><td>Initial</td><td>-61</td><td>-61</td><td>Yes</td><td>1| - |2</td></tr>
<tr><td>Final</td><td>-56</td><td>-56</td><td>Yes</td><td>1 2| - |1 2</td></tr>
<tr><td rowspan="2">B3LYP</td><td>Initial</td><td>-61</td><td>-61</td><td>Yes</td><td>1 2| - |2</td></tr>
<tr><td>Final</td><td>-54</td><td>-55</td><td>Yes</td><td>1 2| - | 1 2</td></tr>
</table>

Table S2 Summary of electronic structure calculations of alkane chain with 30 C atoms in different configurations. The part of the molecule not described by the 10 dihedral angles in the table are in T state. Same notation as in Table S1.

<table>
<thead>
<tr><th></th><th></th><th colspan="10">Dihedral angles</th><th>Local-</th><th></th></tr>
<tr><th>Method</th><th>Geometry</th><th>1</th><th>2</th><th>3</th><th>4</th><th>5</th><th>6</th><th>7</th><th>8</th><th>9</th><th>10</th><th>ize?</th><th>Where?</th></tr>
</thead>
<tbody>
<tr><td></td><td>Start</td><td>G</td><td>G</td><td>G'</td><td>T</td><td>T</td><td>T</td><td>T</td><td>T</td><td>T</td><td>T</td><td></td><td></td></tr>
<tr><td>HF</td><td>Final</td><td>46</td><td>74</td><td>-74</td><td></td><td></td><td></td><td></td><td></td><td></td><td></td><td>Yes</td><td>1 2| - |1 2 3 | -| 2 3</td></tr>
<tr><td>B3LYP-TS</td><td>Final</td><td></td><td></td><td></td><td></td><td></td><td></td><td></td><td></td><td></td><td></td><td></td><td></td></tr>
<tr><td>HF</td><td>B3LYP-TS</td><td></td><td></td><td></td><td></td><td></td><td></td><td></td><td></td><td></td><td></td><td></td><td></td></tr>
<tr><td></td><td>Start</td><td>G</td><td>G'</td><td>G'</td><td>T</td><td>T</td><td>T</td><td>T</td><td>T</td><td>T</td><td>T</td><td></td><td></td></tr>
<tr><td>HF</td><td>Final</td><td>76</td><td>-73</td><td>-59</td><td></td><td></td><td></td><td></td><td></td><td></td><td></td><td>Yes</td><td>1 2 3| - | 1 2 3</td></tr>
<tr><td>B3LYP-TS</td><td>Final</td><td></td><td></td><td></td><td></td><td></td><td></td><td></td><td></td><td></td><td></td><td></td><td></td></tr>
<tr><td>HF</td><td>B3LYP-TS</td><td></td><td></td><td></td><td></td><td></td><td></td><td></td><td></td><td></td><td></td><td></td><td></td></tr>
<tr><td></td><td>Start</td><td>G'</td><td>G'</td><td>G'</td><td>G'</td><td>T</td><td>T</td><td>T</td><td>T</td><td>T</td><td>T</td><td></td><td></td></tr>
<tr><td>HF</td><td>Final</td><td>-67</td><td>-58</td><td>-65</td><td>-80</td><td></td><td></td><td></td><td></td><td></td><td></td><td>Yes</td><td>1 2| -|1 2 3 4|-| 3 4 5 6</td></tr>
<tr><td>B3LYP-TS</td><td>Final</td><td></td><td></td><td></td><td></td><td></td><td></td><td></td><td></td><td></td><td></td><td></td><td></td></tr>
<tr><td>HF</td><td>B3LYP-TS</td><td>-64</td><td>-62</td><td>-58</td><td>-58</td><td></td><td></td><td></td><td></td><td></td><td></td><td>Yes</td><td>1 2| -|1 2 3 4|-| 3 4 5 6</td></tr>
<tr><td></td><td>Start</td><td>G'</td><td>G'</td><td>G'</td><td>G</td><td>T</td><td>T</td><td>T</td><td>T</td><td>T</td><td>T</td><td></td><td></td></tr>
<tr><td>HF</td><td>Final</td><td>-50</td><td>-48</td><td>-83</td><td>77</td><td></td><td></td><td></td><td></td><td></td><td></td><td>Yes/No</td><td>1 2 3 4 5 6|- 1 2 3 4 5 6 - | 1 2 3 4 5 6</td></tr>
<tr><td>B3LYP-TS</td><td>Final</td><td></td><td></td><td></td><td></td><td></td><td></td><td></td><td></td><td></td><td></td><td></td><td></td></tr>
<tr><td>HF</td><td>B3LYP-TS</td><td>-57</td><td>-52</td><td>-72</td><td>75</td><td></td><td></td><td></td><td></td><td></td><td></td><td>Yes/No</td><td>1 2 3 4 5 6| - 1 2 3 4 5 6 - |1 2 3 4 5 6|</td></tr>
<tr><td></td><td>Start</td><td>G'</td><td>G'</td><td>G</td><td>G'</td><td>T</td><td>T</td><td>T</td><td>T</td><td>T</td><td>T</td><td></td><td></td></tr>
<tr><td>HF</td><td>Final</td><td>-45</td><td>-72</td><td>79</td><td>-70</td><td></td><td></td><td></td><td></td><td></td><td></td><td>Yes</td><td>1 2 3 4 5 6|- | 1 2 3 4 5 6| - |1 2 3 4|</td></tr>
<tr><td>B3LYP-TS</td><td>Final</td><td></td><td></td><td></td><td></td><td></td><td></td><td></td><td></td><td></td><td></td><td></td><td></td></tr>
<tr><td>HF</td><td>B3LYP-TS</td><td></td><td></td><td></td><td></td><td></td><td></td><td></td><td></td><td></td><td></td><td></td><td></td></tr>
<tr><td></td><td>Start</td><td>G'</td><td>G</td><td>G'</td><td>G'</td><td>T</td><td>T</td><td>T</td><td>T</td><td>T</td><td>T</td><td></td><td></td></tr>
<tr><td>HF</td><td>Final</td><td>-105</td><td>108</td><td>-65</td><td>-72</td><td></td><td></td><td></td><td></td><td></td><td></td><td>Yes/No</td><td>... 1 2 3 4 5|- |-1 1 2 3 4 5|-|-1 1 2 3 4 5|</td></tr>
<tr><td>B3LYP-TS</td><td>Final</td><td></td><td></td><td></td><td></td><td></td><td></td><td></td><td></td><td></td><td></td><td></td><td></td></tr>
<tr><td>HF</td><td>B3LYP-TS</td><td>-130</td><td>84</td><td>-55</td><td>-66</td><td></td><td></td><td></td><td></td><td></td><td></td><td>No</td><td></td></tr>
<tr><td></td><td>Start</td><td>G</td><td>G'</td><td>G'</td><td>G'</td><td>T</td><td>T</td><td>T</td><td>T</td><td>T</td><td>T</td><td></td><td></td></tr>
<tr><td>HF</td><td>Final</td><td>92</td><td>-58</td><td>-60</td><td>-64</td><td></td><td></td><td></td><td></td><td></td><td></td><td>Yes</td><td>1 2 3| - |3 4</td></tr>
<tr><td>B3LYP-TS</td><td>Final</td><td></td><td></td><td></td><td></td><td></td><td></td><td></td><td></td><td></td><td></td><td></td><td></td></tr>
<tr><td>HF</td><td>B3LYP-TS</td><td></td><td></td><td></td><td></td><td></td><td></td><td></td><td></td><td></td><td></td><td></td><td></td></tr>
<tr><td></td><td>Start</td><td>G</td><td>G</td><td>G'</td><td>G'</td><td>T</td><td>T</td><td>T</td><td>T</td><td>T</td><td>T</td><td></td><td></td></tr>
<tr><td>HF</td><td>Final</td><td>58</td><td>65</td><td>-73</td><td>-64</td><td></td><td></td><td></td><td></td><td></td><td></td><td>Yes</td><td>1 | -|1 2 3 4 | - | 4</td></tr>
<tr><td>B3LYP-TS</td><td>Final</td><td></td><td></td><td></td><td></td><td></td><td></td><td></td><td></td><td></td><td></td><td></td><td></td></tr>
<tr><td>HF</td><td>B3LYP-TS</td><td></td><td></td><td></td><td></td><td></td><td></td><td></td><td></td><td></td><td></td><td></td><td></td></tr>
<tr><td></td><td>Start</td><td>G</td><td>G</td><td>G</td><td>G</td><td>T</td><td>T</td><td>T</td><td>T</td><td>T</td><td>T</td><td></td><td></td></tr>
<tr><td>HF</td><td>Final</td><td>62</td><td>50</td><td>57</td><td>88</td><td></td><td></td><td></td><td></td><td></td><td></td><td>Yes</td><td>1 2| - | 3 4 - |1 2 3 4|</td></tr>
<tr><td>B3LYP-TS</td><td>Final</td><td rowspan="2">63</td><td rowspan="2">62</td><td rowspan="2">58</td><td rowspan="2">58</td><td rowspan="2"></td><td rowspan="2"></td><td rowspan="2"></td><td rowspan="2"></td><td rowspan="2"></td><td rowspan="2"></td><td></td><td></td></tr>
<tr><td>HF</td><td>B3LYP-TS</td><td></td><td></td></tr>
<tr><td></td><td>Start</td><td>G</td><td>G</td><td>G</td><td>G</td><td>G</td><td>T</td><td>T</td><td>T</td><td>T</td><td>T</td><td></td><td></td></tr>
<tr><td>HF</td><td>Final</td><td>60</td><td>67</td><td>60</td><td>58</td><td>51</td><td></td><td></td><td></td><td></td><td></td><td>Yes/No</td><td>|1 2 3 4 5 - 1 2 3 4 5 - |1 2 3 4 5|</td></tr>
<tr><td>B3LYP-TS</td><td>Final</td><td rowspan="2">58</td><td rowspan="2">58</td><td rowspan="2">61</td><td rowspan="2">57</td><td rowspan="2">56</td><td rowspan="2"></td><td rowspan="2"></td><td rowspan="2"></td><td rowspan="2"></td><td rowspan="2"></td><td>Yes</td><td>1 2 3|-|4 5</td></tr>
<tr><td>HF</td><td>B3LYP-TS</td><td>Yes</td><td>1 2 3|-|4 5</td></tr>
<tr><td></td><td>Start</td><td>G</td><td>G</td><td>G</td><td>G</td><td>G</td><td>G</td><td>T</td><td>T</td><td>T</td><td>T</td><td></td><td></td></tr>
<tr><td>HF</td><td>Final</td><td>65</td><td>48</td><td>53</td><td>80</td><td>70</td><td>53</td><td></td><td></td><td></td><td></td><td>Yes</td><td>1 2 3 4 5|- |1 2 3 4 5 6|- 1 2| |56</td></tr>
<tr><td>B3LYP-TS</td><td>Final</td><td rowspan="2">58</td><td rowspan="2">55</td><td rowspan="2">58</td><td rowspan="2">55</td><td rowspan="2">52</td><td rowspan="2">57</td><td rowspan="2"></td><td rowspan="2"></td><td rowspan="2"></td><td rowspan="2"></td><td>Yes</td><td>1 2 3 4 5| - |3 4 5 6 - |1 2 3 4 5 6|</td></tr>
<tr><td>HF</td><td>B3LYP-TS</td><td>Yes</td><td>1 2 3 4 5| - |3 4 5 6 - |1 2 3 4 5 6|</td></tr>
<tr><td></td><td>Start</td><td>G</td><td>G</td><td>G</td><td>G</td><td>G</td><td>G</td><td>G</td><td>T</td><td>T</td><td>T</td><td></td><td></td></tr>
<tr><td>HF</td><td>Final</td><td>57</td><td>75</td><td>52</td><td>79</td><td>52</td><td>55</td><td>54</td><td></td><td></td><td></td><td>Yes</td><td>..1|-|1 2 3 4 5 6 7|-|7 ...</td></tr>
<tr><td>B3LYP-TS</td><td>Final</td><td rowspan="2">56</td><td rowspan="2">56</td><td rowspan="2">58</td><td rowspan="2">59</td><td rowspan="2">61</td><td rowspan="2">62</td><td rowspan="2">55</td><td rowspan="2"></td><td rowspan="2"></td><td rowspan="2"></td><td>No</td><td></td></tr>
<tr><td>HF</td><td>B3LYP-TS</td><td>Yes/No</td><td>..1|-|1 2 3 4 5 6 7|-|7 ...</td></tr>
<tr><td></td><td>Start</td><td>G</td><td>G</td><td>G</td><td>G</td><td>G</td><td>G</td><td>G</td><td>G</td><td>T</td><td>T</td><td></td><td></td></tr>
<tr><td>HF</td><td>Final</td><td>66</td><td>65</td><td>68</td><td>49</td><td>52</td><td>66</td><td>53</td><td>56</td><td></td><td></td><td>Yes</td><td>..1|-|1 2 3 4 5 6 7 8|-|8 ...</td></tr>
<tr><td>B3LYP-TS</td><td>Final</td><td rowspan="2">64</td><td rowspan="2">65</td><td rowspan="2">57</td><td rowspan="2">51</td><td rowspan="2">53</td><td rowspan="2">64</td><td rowspan="2">64</td><td rowspan="2">57</td><td rowspan="2"></td><td rowspan="2"></td><td>No</td><td></td></tr>
<tr><td>HF</td><td>B3LYP-TS</td><td>Yes/No</td><td>..1|-|1 2 3 4 5 6 7 8|-|8 ...</td></tr>
<tr><td></td><td>Start</td><td>G</td><td>G</td><td>G</td><td>G</td><td>G</td><td>G</td><td>G</td><td>G</td><td>G</td><td>T</td><td></td><td></td></tr>
<tr><td>HF</td><td>Final</td><td>72</td><td>52</td><td>72</td><td>68</td><td>76</td><td>59</td><td>62</td><td>60</td><td>69</td><td></td><td>Yes</td><td>..1|-|1 2 3 4 5 6 7 8 9|-|9 ...</td></tr>
<tr><td>B3LYP-TS</td><td>Final</td><td rowspan="2">67</td><td rowspan="2">62</td><td rowspan="2">65</td><td rowspan="2">58</td><td rowspan="2">56</td><td rowspan="2">59</td><td rowspan="2">61</td><td rowspan="2">60</td><td rowspan="2">64</td><td rowspan="2"></td><td>Yes</td><td>..1|-|1 2 3 4 5 6 7 8 9|-|9 ...</td></tr>
<tr><td>HF</td><td>B3LYP-TS</td><td>Yes/No</td><td>..1 9...|-|1 2 3 4 5 6 7 8 9|-|... 1 9 ...</td></tr>
<tr><td></td><td>Start</td><td>G</td><td>G</td><td>G</td><td>G</td><td>G</td><td>G</td><td>G</td><td>G</td><td>G</td><td>G</td><td></td><td></td></tr>
<tr><td>HF</td><td>Final</td><td>45</td><td>61</td><td>55</td><td>60</td><td>63</td><td>70</td><td>59</td><td>64</td><td>69</td><td>78</td><td>Yes/No</td><td>..1|-|1 2 3 4 5 6 7 8 9 10|-|10 ...</td></tr>
<tr><td>B3LYP-TS</td><td>Final</td><td rowspan="2">56</td><td rowspan="2">58</td><td rowspan="2">64</td><td rowspan="2">63</td><td rowspan="2">57</td><td rowspan="2">58</td><td rowspan="2">56</td><td rowspan="2">63</td><td rowspan="2">70</td><td rowspan="2">71</td><td>Yes</td><td>..1|-|1 2 3 4 5 6 7 8 9 10|-|10 ...</td></tr>
<tr><td>HF</td><td>B3LYP-TS</td><td>Yes/No</td><td>..1|-|1 2 3 4 5 6 7 8 9 10|-|10 ...</td></tr>
</tbody>
</table>

Table S3 Summary of electronic structure calculations of alkane chain with 30 C atoms in different configurations. The part of the molecule not described by the 10 dihedral angles in the table are in T state. Same notation as in Table S1.

<table>
<tr><th></th><th></th><th colspan="10">Dihedral angles</th><th></th><th></th></tr>
<tr><th>Method</th><th>Geometry</th><th>1</th><th>2</th><th>3</th><th>4</th><th>5</th><th>6</th><th>7</th><th>8</th><th>9</th><th>10</th><th>Localize?</th><th>Where?</th></tr>
<tr><td></td><td>Start</td><td>T</td><td>T</td><td>G</td><td>T</td><td>G</td><td>T</td><td>T</td><td>T</td><td>T</td><td>T</td><td></td><td></td></tr>
<tr><td>HF</td><td>Final</td><td>175</td><td>179</td><td>59</td><td>-174</td><td>49</td><td>169</td><td>-178</td><td>180</td><td></td><td></td><td>Yes</td><td>1 2 3|-|3 .. 7</td></tr>
<tr><td>B3LYP-TS</td><td>Final</td><td rowspan="2">180</td><td rowspan="2">173</td><td rowspan="2">63</td><td rowspan="2">180</td><td rowspan="2">61</td><td rowspan="2">172</td><td rowspan="2">178</td><td rowspan="2">179</td><td rowspan="2"></td><td rowspan="2"></td><td>Yes</td><td>1 2 3|-|3 .. 7</td></tr>
<tr><td>HF</td><td>B3LYP-TS</td><td>Yes</td><td>1 2 3|-|3 .. 7</td></tr>
<tr><td></td><td>Start</td><td>T</td><td>T</td><td>G</td><td>T</td><td>T</td><td>G</td><td>T</td><td>T</td><td>T</td><td>T</td><td></td><td></td></tr>
<tr><td>HF</td><td>Final</td><td></td><td>179</td><td>61</td><td>179</td><td>180</td><td>61</td><td>179</td><td></td><td></td><td></td><td>Yes</td><td>2 .. 5|-|6 7</td></tr>
<tr><td>B3LYP-TS</td><td>Final</td><td rowspan="2"></td><td rowspan="2">176</td><td rowspan="2">55</td><td rowspan="2">174</td><td rowspan="2">171</td><td rowspan="2">60</td><td rowspan="2">178</td><td rowspan="2"></td><td rowspan="2"></td><td rowspan="2"></td><td>Yes</td><td>2 .. 5|-|5 6 7</td></tr>
<tr><td>HF</td><td>B3LYP-TS</td><td>Yes</td><td>2 .. 5|-|6 7</td></tr>
<tr><td></td><td>Start</td><td>T</td><td>T</td><td>G</td><td>T</td><td>T</td><td>T</td><td>G</td><td>T</td><td>T</td><td>T</td><td></td><td></td></tr>
<tr><td>HF</td><td>Final</td><td>177</td><td>-177</td><td>82</td><td>176</td><td>-179</td><td>-174</td><td>83</td><td>-176</td><td></td><td></td><td>No</td><td></td></tr>
<tr><td>B3LYP-TS</td><td>Final</td><td rowspan="2">180</td><td rowspan="2">-176</td><td rowspan="2">73</td><td rowspan="2">-179</td><td rowspan="2">-177</td><td rowspan="2">180</td><td rowspan="2">67</td><td rowspan="2">179</td><td rowspan="2"></td><td rowspan="2"></td><td>Yes/No</td><td>1 2 3|-|3 .. 7|-|7 8</td></tr>
<tr><td>HF</td><td>B3LYP-TS</td><td>Yes/No</td><td>1 2 3|-|3 .. 7|-|7 8</td></tr>
<tr><td></td><td>Start</td><td>T</td><td>G</td><td>T</td><td>T</td><td>T</td><td>T</td><td>G</td><td>T</td><td>T</td><td>T</td><td></td><td></td></tr>
<tr><td>HF</td><td>Final</td><td>168</td><td>58</td><td>174</td><td>172</td><td>171</td><td>172</td><td>55</td><td>180</td><td>180</td><td></td><td>Yes</td><td>1 2|-|2 .. 7|-|7 8 9</td></tr>
<tr><td>B3LYP-TS</td><td>Final</td><td rowspan="2">177</td><td rowspan="2">56</td><td rowspan="2">173</td><td rowspan="2">175</td><td rowspan="2">172</td><td rowspan="2">174</td><td rowspan="2">56</td><td rowspan="2">171</td><td rowspan="2">-179</td><td rowspan="2"></td><td>Yes</td><td>1 2|-|2 .. 7|-|7 8 9</td></tr>
<tr><td>HF</td><td>B3LYP-TS</td><td></td><td></td></tr>
<tr><td></td><td>Start</td><td>G</td><td>T</td><td>T</td><td>T</td><td>T</td><td>T</td><td>G</td><td>T</td><td>T</td><td>T</td><td></td><td></td></tr>
<tr><td>HF</td><td>Final</td><td></td><td></td><td></td><td></td><td></td><td></td><td></td><td></td><td></td><td></td><td></td><td></td></tr>
<tr><td>B3LYP-TS</td><td>Final</td><td rowspan="2">57</td><td rowspan="2">-178</td><td rowspan="2">176</td><td rowspan="2">-173</td><td rowspan="2">174</td><td rowspan="2">175</td><td rowspan="2">57</td><td rowspan="2">174</td><td rowspan="2">178</td><td rowspan="2"></td><td>Yes</td><td>1|-|1 .. 8|-| 8 9</td></tr>
<tr><td>HF</td><td>B3LYP-TS</td><td>Yes</td><td>1| |8 9 - |1 .. 8|-| 8[illegible]</td></tr>
<tr><td></td><td>Start</td><td>G</td><td>T</td><td>T</td><td>T</td><td>T</td><td>T</td><td>T</td><td>G</td><td>T</td><td>T</td><td></td><td></td></tr>
<tr><td>HF</td><td>Final</td><td></td><td></td><td></td><td></td><td></td><td></td><td></td><td></td><td></td><td></td><td></td><td></td></tr>
<tr><td>B3LYP-TS</td><td>Final</td><td rowspan="2">55</td><td rowspan="2">171</td><td rowspan="2">178</td><td rowspan="2">175</td><td rowspan="2">175</td><td rowspan="2">175</td><td rowspan="2">-174</td><td rowspan="2">64</td><td rowspan="2">173</td><td rowspan="2"></td><td>Yes</td><td>1|-|1 .. 8|-| 8 9</td></tr>
<tr><td>HF</td><td>B3LYP-TS</td><td>Yes</td><td>1|-|1 .. 8|-| 8 9</td></tr>
<tr><td></td><td>Start</td><td>G</td><td>T</td><td>T</td><td>T</td><td>T</td><td>T</td><td>T</td><td>T</td><td>G</td><td>T</td><td></td><td></td></tr>
<tr><td>HF</td><td>Final</td><td></td><td></td><td></td><td></td><td></td><td></td><td></td><td></td><td></td><td></td><td></td><td></td></tr>
<tr><td>B3LYP-TS</td><td>Final</td><td rowspan="2">59</td><td rowspan="2">179</td><td rowspan="2">176</td><td rowspan="2">-177</td><td rowspan="2">177</td><td rowspan="2">-177</td><td rowspan="2">-178</td><td rowspan="2">-179</td><td rowspan="2">75</td><td rowspan="2"></td><td>No</td><td></td></tr>
<tr><td>HF</td><td>B3LYP-TS</td><td>Yes/No</td><td>1.. 9|-1| |9 - |1..9|</td></tr>
<tr><td></td><td>Start</td><td>G</td><td>T</td><td>T</td><td>T</td><td>T</td><td>T</td><td>T</td><td>T</td><td>T</td><td>G</td><td></td><td></td></tr>
<tr><td>HF</td><td>Final</td><td></td><td></td><td></td><td></td><td></td><td></td><td></td><td></td><td></td><td></td><td></td><td></td></tr>
<tr><td>B3LYP-TS</td><td>Final</td><td rowspan="2">66</td><td rowspan="2">171</td><td rowspan="2">180</td><td rowspan="2">175</td><td rowspan="2">-179</td><td rowspan="2">178</td><td rowspan="2">-178</td><td rowspan="2">-176</td><td rowspan="2">-174</td><td rowspan="2">82</td><td>No</td><td></td></tr>
<tr><td>HF</td><td>B3LYP-TS</td><td>No</td><td></td></tr>
</table>

A. Additional illustrations

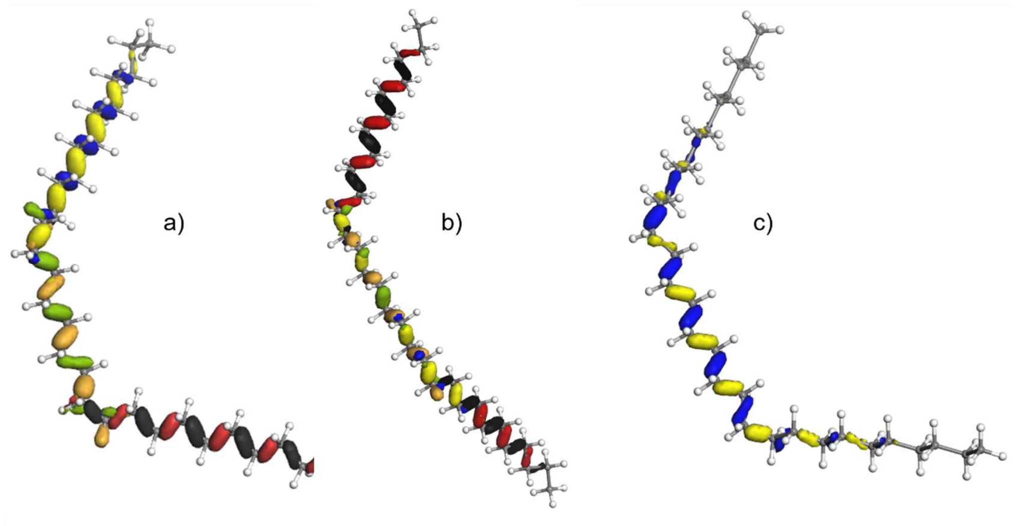

Figure S1 *Orbitals from HF calculations. HOMO (blue-yellow), HOMO-1 (black-red) and HOMO-2 (orange-green), for different configurations of the mid section of the alkane chain. a) TG(6T)GT b)TG(7T)GT and c) TG(8T)GT.*

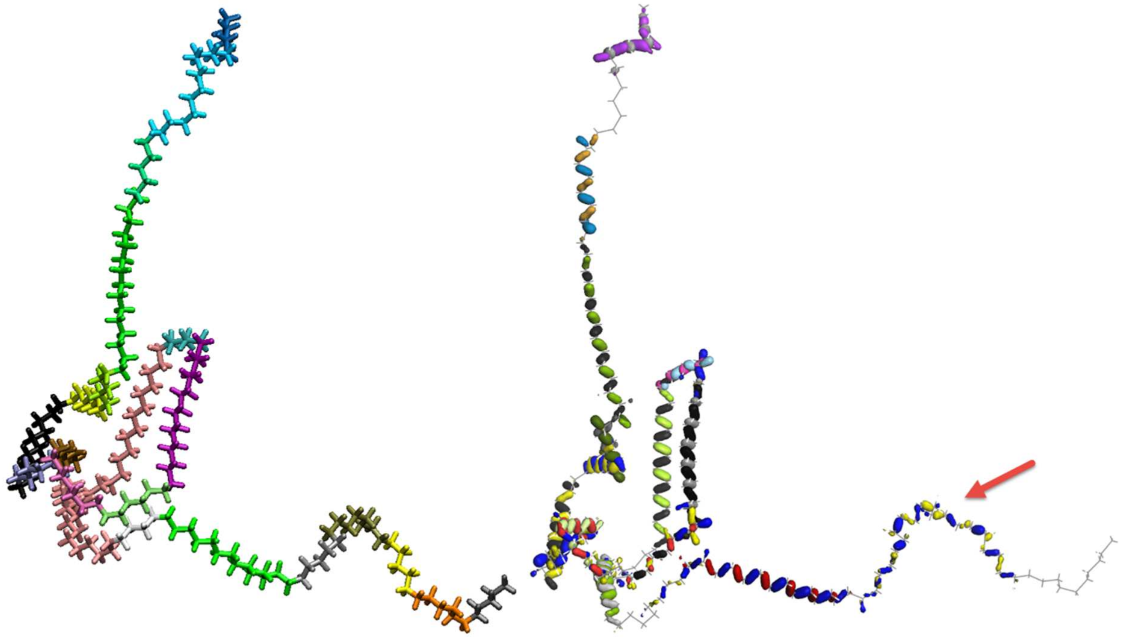

Figure S2 *A single chain from the amorphous PE model. Left: Segments obtained following the method in this work, the color indicates the separate segments, in total 22. Right: The 19 orbitals from BLYP-TS calculations. About 4 parts of the polymer has none of the 19 orbitals localized to these parts. The arrow shows a part of the polymer where the segmentation and orbital localization does not seem to match.*

II. SITE DUPLEXES

The derivation of the site duplex round-trip reduction algorithm from Ref. 1 is here reproduced with some minor differences to match text in the main text of this article.

An easy way to improve performance is to model the repeated transitions between A and B using the expected number of round-trips, rather than directly simulating each hop in each round trip.

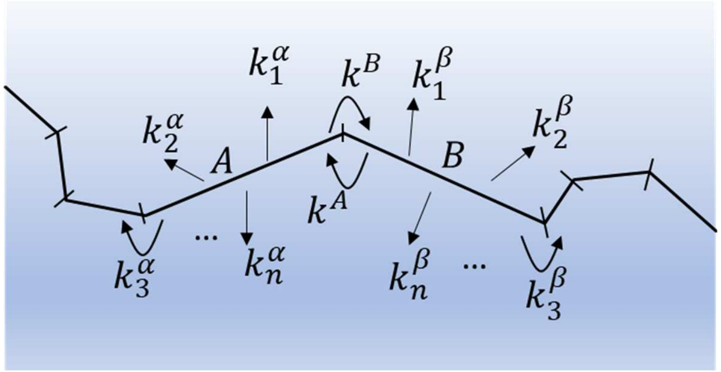

Figure S3 Illustration of two adjacent sites A and B along a polymer chain forming a duplex. The hopping rates k^A and k^B are much higher compared to the other hopping rates, k_i^α and k_i^β $i = 1 \dots n$.

A. Expected dwell time

We first note that the dwell time in site A or B is independent of hop destination, and thus the time spent at each site can be calculated without knowledge of the next hop. Applying the dwell time expression for KMC on the duplex of the system in Figure S1 gives the expected dwell times as

$$\tau_A = \mathbb{E}\left(-\frac{1}{\sum_i k_i^\alpha + k^B}\ln \mathcal{U}(0,1)\right) = \frac{1}{\sum_i k_i^\alpha + k^B} \tag{1}$$

and

$$\tau_B = \mathbb{E}\left(-\frac{1}{\sum_i k_i^\beta + k^A}\ln \mathcal{U}(0,1)\right) = \frac{1}{\sum_i k_i^\beta + k^A} \tag{2}$$

where τ_A and τ_B are the expected dwell times at site A and B, respectively,

and $\tau_{AB} = \tau_A + \tau_B$ is the expected round-trip time for the transition A → B → A.

B. Exiting the duplex

Consider a charge carrier entering at site A in figure 3.5 and performing a certain number of round-trips between A and B before exiting the A-B duplex. The carrier is considered to have exited the duplex when it hops to a site in either $\{\alpha_i\}$ or $\{\beta_i\}$. A hop to $\{\alpha_i\}$ is considered an exit from A, whereas a hop to $\{\beta_i\}$ is considered an exit from B. Since hop probability is proportional to hopping rate, the probability to jump to a certain exit site conditional on an exit from either A or B is straightforward

$$\mathbb{P}(A \to \alpha_i|\text{Exit from A}) = \frac{k_i^\alpha}{\sum_j k_j^\alpha} \tag{3}$$

and

$$\mathbb{P}(B \to \beta_i | \text{Exit from B}) = \frac{k_i^\beta}{\sum_j k_j^\beta} \quad (4)$$

These conditional probabilities can be further used to get the general probability of the carrier hopping to a certain exit site after entering the duplex:

$$\mathbb{P}(\text{Exit to } \alpha_i) = \mathbb{P}(\text{Exit from A})\mathbb{P}(A \to \alpha_i | \text{Exit from A}) \quad (5)$$

and

$$\mathbb{P}(\text{Exit to } \beta_i) = \mathbb{P}(\text{Exit from B})\mathbb{P}(B \to \beta_i | \text{Exit from B}) \quad (6)$$

where $\mathbb{P}(\text{Exit from A}) = 1 - \mathbb{P}(\text{Exit from B})$ by definition.

Now let *t* be the total dwell time inside the duplex, i.e. the total time between the entry and the exit of the charge carrier. This dwell time will in general depend on whether the carrier exits from A or B. Furthermore, let *r* be the number of round-trips A → B → A made before exiting the duplex. Using the previously derived expected dwell times for A and B, the expected total dwell time inside the duplex can be defined as

$$\mathbb{E}(t | \text{Exit from A}) = \mathbb{E}(r | \text{Exit from A})\tau_{AB} + \tau_A \quad (7)$$

and

$$\mathbb{E}(t | \text{Exit from B}) = \mathbb{E}(r | \text{Exit from B})\tau_{AB} + \tau_A + \tau_B \quad (8)$$

which takes into account the expected time spent for each round-trip and the expected time spent on one or two non round-trip hops required to exit the system. Missing from these calculations are the probability $\mathbb{P}(\text{Exit from A})$ and expected values $\mathbb{E}(r | \text{Exit from A})$ and $\mathbb{E}(r | \text{Exit from B})$ which will be calculated separately below.

C. Exit probability

From KMC we know that the probability of a charge carrier in A hopping to B as its next hop, and vice versa, is

$$\mathbb{P}(A \to \text{B}) = \frac{k^B}{\sum_i k_i^\alpha + k^B} \quad (9)$$

and

$$\mathbb{P}(B \to A) = \frac{k^A}{\sum_i k_i^\beta + k^A} \quad (10)$$

with complementary probabilities

$$\mathbb{P}(A \to \{\alpha_i\}) = 1 - \mathbb{P}(A \to B) \quad (11)$$

and

$$\mathbb{P}(B \to \{\beta_i\}) = 1 - \mathbb{P}(B \to A) \quad (12)$$

Furthermore, the probability of a carrier in A performing a round-trip and returning to A in two hops is

$$\mathbb{P}(A \to B \to A) = \mathbb{P}(A \to B)\mathbb{P}(B \to A) \quad (13)$$

The probability of the carrier exiting via A but performing no round-trips is $\mathbb{P}(A \to \{\alpha_i\})$. The probability of the carrier performing exactly x round trips and then exiting A is

$\mathbb{P}(A \to B \to A)^n \mathbb{P}(A \to \{\alpha_i\})$. From this the probability of the carrier exiting via A after an arbitrary number of round-trips can be defined as a convergent infinite geometric series

$$\begin{aligned} \mathbb{P}(\text{Exit from } A) &= \mathbb{P}(A \to \{\alpha_i\}) \sum_{n}^{\infty} \mathbb{P}(A \to B \to A)^n \\ &= \frac{\mathbb{P}(A \to \{\alpha_i\})}{1 - \mathbb{P}(A \to B \to A)} \end{aligned} \tag{14}$$

D. Expected number of round-trips

As in the previous section, the probability of a charge carrier in A performing n round-trips before exiting the system from A is $\mathbb{P}(A \to B \to A)^n \mathbb{P}(A \to \{\alpha_i\})$. From this the expected number of round trips $\mathbb{E}(r|\text{Exit from A})$ can be calculated as an arithmetico-geometric sequence

$$\begin{aligned} \mathbb{E}(r|\text{Exit from A}) &= \frac{\sum_{n=0}^{\infty} n\mathbb{P}(r = n \cup \text{Exit from A})}{\mathbb{P}(\text{Exit from A})} \\ &= \frac{\mathbb{P}(A \to \{\alpha_i\})}{\mathbb{P}(\text{Exit from A})} \sum_{n=0}^{\infty} n\mathbb{P}(A \to B \to A)^n \\ &= \frac{\mathbb{P}(A \to \{\alpha_i\})}{\mathbb{P}(\text{Exit from A})} \frac{\mathbb{P}(A \to B \to A)}{\left(1 - \mathbb{P}(A \to B \to A)\right)^2} = \frac{\mathbb{P}(A \to B \to A)}{1 - \mathbb{P}(A \to B \to A)} \end{aligned} \tag{15}$$

The expected value $\mathbb{E}(r|\text{Exit from B})$ can be calculated in an equivalent manner

$$\mathbb{E}(r|\text{Exit from B}) = \frac{\mathbb{P}(A \to \{\beta_i\})}{\mathbb{P}(\text{Exit from B})} \frac{\mathbb{P}(A \to B \to A)}{\left(1 - \mathbb{P}(A \to B \to A)\right)^2} = \frac{\mathbb{P}(A \to B \to A)}{1 - \mathbb{P}(A \to B \to A)} \tag{16}$$

E. Final algorithm

Using these building blocks, an extension of the KMC algorithm was constructed wherein the effect of round-trips between A and B on mobility was taken into account without necessitating spending simulation steps on each individual hop. Whenever the charge carrier enters the strongly interconnected duplex via A:

- Choose the next hopping site from among $\{\alpha_i\}$ and $\{\beta_i\}$ according to probabilities $\mathbb{P}(\text{Exit to } \alpha_i)$ and $\mathbb{P}(\text{Exit to } \beta_i)$ as defined in equation (5) and (6).
- Associate any hop to $\{\alpha_i\}$ and $\{\beta_i\}$ with a duplex dwell time $\mathbb{E}(t|\text{Exit from A})$ and $\mathbb{E}(t|\text{Exit from B})$, respectively, as defined in equation (7) and (8).

The equivalent calculations for a carrier entering the duplex via B can be recovered by swapping A and B together with α and β. It would be straightforward to extend the algorithm by sampling the duplex dwell time t from an equivalent probability distribution, but for this study the mean dwell time is adequate since the focus is the average mobility across a very large number of hops.

III. References

(1) Aspåker, H. Study of hole mobility in amorphous polyethylene via kinetic Monte Carlo methods. MSc thesis, KTH Royal Insitute of Technology, 2023.
